# Identification of two novel powdery mildew resistance loci, *Ren6* and *Ren7*, from the wild Chinese grape species *Vitis piasezkii*

**DOI:** 10.1186/s12870-016-0855-8

**Published:** 2016-07-29

**Authors:** Dániel Pap, Summaira Riaz, Ian B. Dry, Angelica Jermakow, Alan C. Tenscher, Dario Cantu, Róbert Oláh, M. Andrew Walker

**Affiliations:** 1 Department of Viticulture and Enology, University of California, Davis, CA 95616 USA; 2 CSIRO Agriculture, Glen Osmond, SA Australia; 3 Department of Genetics and Plant Breeding, Corvinus University of Budapest, Villányi út 29-34, 1118 Budapest, Hungary

**Keywords:** Disease resistance, Powdery mildew, Grape breeding, *Vitis piasezkii*, *Ren6*, *Ren7*

## Abstract

**Background:**

Grapevine powdery mildew *Erysiphe necator* is a major fungal disease in all grape growing countries worldwide. Breeding for resistance to this disease is crucial to avoid extensive fungicide applications that are costly, labor intensive and may have detrimental effects on the environment. In the past decade, Chinese *Vitis* species have attracted attention from grape breeders because of their strong resistance to powdery mildew and their lack of negative fruit quality attributes that are often present in resistant North American species. In this study, we investigated powdery mildew resistance in multiple accessions of the Chinese species *Vitis piasezkii* that were collected during the 1980 Sino-American botanical expedition to the western Hubei province of China.

**Results:**

A framework genetic map was developed using simple sequence repeat markers in 277 seedlings of an F1 mapping population arising from a cross of the powdery mildew susceptible *Vitis vinifera* selection F2-35 and a resistant accession of *V. piasezkii* DVIT2027. Quantitative trait locus analyses identified two major powdery mildew resistance loci on chromosome 9 (*Ren6*) and chromosome 19 (*Ren7*) explaining 74.8 % of the cumulative phenotypic variation. The quantitative trait locus analysis for each locus, in the absence of the other, explained 95.4 % phenotypic variation for *Ren6*, while *Ren7* accounted for 71.9 % of the phenotypic variation. Screening of an additional 259 seedlings of the F1 population and 910 seedlings from four pseudo-backcross populations with SSR markers defined regions of 22 kb and 330 kb for *Ren6* and *Ren7* in the *V. vinifera* PN40024 (12X) genome sequence, respectively.

Both *R* loci operate post-penetration through the induction of programmed cell death, but vary significantly in the speed of response and degree of resistance; *Ren6* confers complete resistance whereas *Ren7* confers partial resistance to the disease with reduced colony size. A comparison of the kinetics of induction of powdery mildew resistance mediated by *Ren6*, *Ren7* and the *Run1* locus from *Muscadinia rotundifolia*, indicated that the speed and strength of resistance conferred by *Ren6* is greater than that of *Run1* which, in turn, is superior to that conferred by *Ren7*.

**Conclusions:**

This is the first report of mapping powdery mildew resistance in the Chinese species *V. piasezkii*. Two distinct powdery mildew *R* loci designated *Ren6* and *Ren7* were found in multiple accessions of this Chinese grape species. Their location on different chromosomes to previously reported powdery mildew resistance *R* loci offers the potential for grape breeders to combine these *R* genes with existing powdery mildew *R* loci to produce grape germplasm with more durable resistance against this rapidly evolving fungal pathogen.

**Electronic supplementary material:**

The online version of this article (doi:10.1186/s12870-016-0855-8) contains supplementary material, which is available to authorized users.

## Background

Grapevine powdery mildew (PM) is caused by the obligate biotrophic fungus *Erysiphe necator* Schwein. (previously *Uncinula necator*). The widely cultivated European grape, *Vitis vinifera* L., is highly susceptible to powdery mildew. All green plant parts suffer from infection resulting in reduced yield due to a decline of leaf photosynthetic capacity and compromised fruit and wine quality [1–3]. Complete crop loss can occur if infection is not controlled in the early stages of flowering and fruit set. A rigorous regime of synthetic and organic fungicide applications with seasonal rotations (as many as 12 to 20 times in one growing season) is required to control the disease and to prevent the pathogen from evolving resistance to fungicides. Excessive application of fungicides leads to increased production costs and adverse impacts on the environment [4–6].

Breeding grape varieties to resist powdery mildew is a direct strategy to increase farming efficiency and reduce the environmental impact of frequent prophylactic fungicide sprays. Many North American species have natural resistance to powdery mildew and a number of *R* loci have been identified for this fungal disease (*Run1* [7, 8]; *Run2.1*, *Run2.2* [9]; *Ren2* [10]; *Ren3* [11]; *Ren5* [12]). Many of these *R* loci are being used in different breeding programs [13, 14].

In the last decade, wild germplasm in Asia gained the attention of grape breeders due to the presence of high levels of resistance to powdery mildew [15]. Chinese species hybridize easily, and lack the negative fruit properties present in the hybrids of North American species, which make them prime candidates for breeding [16]. Among the approximately 35 Asian *Vitis* species, powdery mildew resistance-linked *R* loci have previously been identified and mapped in two full-sib accessions (C166-043 and C166-026) of *V. romanetii* (*Ren4* [9, 17, 18]. Moreover, the *Ren1* locus that provides partial resistance to powdery mildew was also identified in two accessions of cultivated *V. vinifera* from central Asia [19, 20]. In an attempt to explore new potential sources of PM resistance in central Asian accessions, Riaz et al. [21] surveyed 380 cultivated and wild accessions of *V. vinifera* and accessions of Chinese species. They identified 10 *V. vinifera* accessions with partial resistance to PM and strong resistance in accessions of the Chinese species, *V. piasezkii*. Powdery mildew resistance has previously been reported in multiple accessions of *V. piasezkii*, a species widely distributed in the wild grape species rich mountain ranges of Northeast and Western China [15, 22].

It is a general assumption that PM originated from North America based on the historical records and presence of resistance in many North American species [5, 23]. The presence of strong resistance to powdery mildew in Chinese *Vitis* species is curious. Potentially, these Chinese species could have different mechanisms of resistance at the molecular level. From a breeding perspective, it is critical to combine *R* loci that recognize different molecular patterns into the same genotype to generate durable field resistance that is not overcome by rapidly evolving pathogens [14, 24–26]. This approach should consider the combination of different host resistance mechanisms and the knowledge of powdery mildew core effectors recognized by different *R* genes. Understanding of the resistance will greatly assist breeders in making decisions about combining different loci to develop breeding lines with durable resistance in the field [27]. Enhancing genetic resistance of cultivated grapevines would potentially lead to powdery mildew management with reduced or no fungicide applications, lowering costs of production and reducing the impact on the natural environment [5, 27–29].

In this study we investigated powdery mildew resistance in ten accessions of *V. piasezkii* maintained at the National Clonal Germplasm Repository, Davis, California. Nine of these accessions were collected during the 1980 Sino-American botanical expedition in the Shennongjia Forestry District, Hubei province of China [30, 31]. Two accessions DVIT2027 and DVIT2032 were identified to be powdery mildew resistant in an earlier study [21]. The DVIT2027 accession was used to develop two F1 breeding populations; a framework genetic map with simple sequence repeat (SSR) markers was developed and two new powdery mildew *R* loci on different chromosomes were identified. These loci have been designated *Ren6* (chromosome 9) and *Ren7* (chromosome 19) in accordance with the guidelines of the International Grape Genome Program [32]. The large population size allowed us to study the effect of each locus cumulatively as well as individually. The *Ren6* locus provides complete immunity to the disease by initiating rapid programmed cell death (PCD) at the point of pathogen penetration. The locus *Ren7* provides partial resistance by allowing the pathogen to establish, but limits the amount of hyphal growth and conidiation. The availability of these two new *R* loci will enhance the repertoire of existing *R* loci *available* for powdery mildew resistance breeding.

## Methods

### Plant material

The F1 population designated 11-373 was the result of a cross between powdery mildew susceptible and pistillate *V. vinifera* F2-35 (‘Carignane’ × ‘Cabernet Sauvignon’) and the resistant Chinese species *V. piasezkii* DVIT2027. This accession of *V. piasezkii* was identified to have strong resistance to grape powdery mildew in multi-year field-testing and it hybridizes easily with other *Vitis* species making it an excellent parent for powdery mildew resistance breeding. DVIT2027 is a staminate vine; leaves are simple, unlobed and long cordate with an acute apex. This accession does not have shoots with variable leaf lobing, such irregular lobing is typical of *V. piasezkii*.

**Fig. 1:**
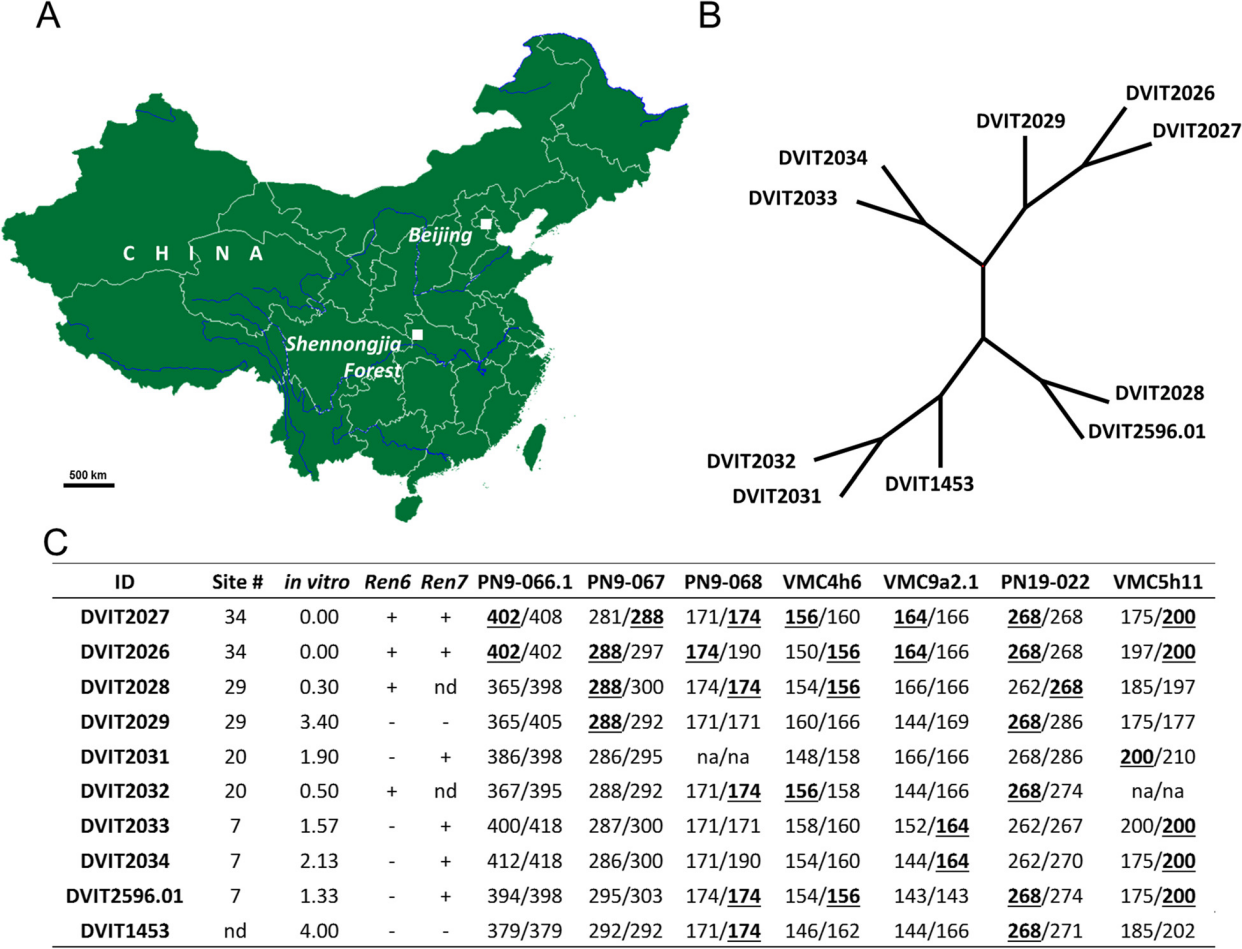
Identification of *Ren6* and *Ren7* loci with interval and multiple QTL mapping. (**a**, **b**) Results of interval mapping carried out on the mapping population for chromosome 9 and 19. (**c**, **d**) Interval mapping analysis on subset of genotypes selected on the basis of the local haplotype of *Ren6* or *Ren7* locus, respectively. (**e**) Results of Multiple QTL mapping on the 19 chromosomes of DVIT2027. Leaf 2013, Leaf 2014 and Cane 2013, Cane 2014 represent the disease evaluations carried out in the field for the respective year. Greenhouse and in vitro assays were carried out in controlled environments. In all charts the arrow represents the maximum LOD score and the respective percent-explained variation of the greenhouse screen for powdery mildew resistance. The *red dotted line* represents the significance threshold for QTL detection

The 11-373 breeding population consisted of 536 seedlings that are maintained at the Department of Viticulture and Enology, University of California, Davis, California. The DVIT2027 and eight other accessions of *V. piasezkii* were collected in 1980 during the Sino-American Botanical expedition (Fig. 1) [30]. The accession DVIT1453 was acquired from China by H. P. Olmo. All accessions of *V. piasezkii* tested in this study are maintained at the USDA-ARS National Clonal Germplasm Repository, Davis, California.

An additional small F1 mapping population (designated VpF1) was generated by crossing *V. piasezkii* DVIT2027 with a powdery mildew-susceptible *V. vinifera* ‘Pinot Meunier’ mutant “picovine” line 06C008V0003 [33]. The VpF1 population consisting of 31 individuals was maintained in a glasshouse at CSIRO Agriculture, Urrbrae, South Australia. This process was similar to how the *Run1* locus from *M. rotundifolia* was introduced into the same genetic background by crossing the resistant line BC5:3294-R23 with picovine line 06C008V0003 and selecting *Run1* progeny using markers as described previously [34].

### Disease evaluations

The 11-373 seedling population was evaluated for powdery mildew resistance in multiple environments. Severity of the disease symptoms was recorded in two successive years under natural and artificial infections in the field. Disease evaluations were also carried out on four replicates of each seedling plant in a controlled environment in an unsprayed greenhouse, and by detached leaf assay in the laboratory. The powdery mildew mass was quantified on detached leaf samples with a molecular approach using quantitative polymerase chain reaction (qPCR).

Powdery mildew symptoms on canes and leaves were evaluated on all available growing plants in the field. From the base mapping population of 277 seedlings, 253 and 261 seedlings were evaluated in the field in 2013 and 2014, respectively. A total of 258 seedlings from the base population were challenged with powdery mildew and evaluated in the greenhouse. Young uninfected leaves of 258 seedlings were also used in an in vitro assay and examined under the microscope (Leica EZ4 D) for severity of mildew infection. To avoid bias, plants in the field were scored three to four times each year and two people independently evaluated the greenhouse and the in vitro experiments. Lastly, qPCR was completed on 247 genotypes from the in vitro assay leaves to measure the total mass of fungal infection.

Categorical measurements of phenotypic responses in the field were recorded in August, September and October of 2013 under natural infection conditions. In the spring of 2014 artificial inoculations were carried out at four subsequent times from April to the end of June with 3 to 4 week intervals in order to ensure a homogeneously high infection rate. For artificial inoculations, PM conidia were amplified on in vitro cultures of *V. vinifera* ‘Carignane’ leaves and suspended in 0.1 % (v/v) Tween-20 solution. Each seedling plant was sprayed with the inoculum suspension using a Perval Sprayer unit (Chicago Aerosol, Coal City, Illinois). The powdery mildew symptoms were evaluated in August and September. A 6 point scoring system was used for both leaf and cane scores in years 2013 and 2014: 0 - no visible symptoms, 1 - one or two spots of infection, 2 - more than two spots of infection but still hard to find, 3 - active PM infection that was easy to observe on the leaves and cane tissue, 4 - PM infection patches on many leaves and cane tissue, and 5 - heavy PM infection on all plant parts.

Controlled disease evaluations were performed in an unsprayed shaded greenhouse and on detached leaves in the laboratory. For the greenhouse evaluations, three to four replicates of each genotype were propagated from either green or hardwood cuttings and potted in 10 cm pots. Multiple plants of susceptible control cultivars (*V. vinifera* ‘Carignane’ and F2-35), and tolerant/resistant controls (*V. vinifera* ‘Karadzhandal’, *Vitis* hybrids ‘Villard Blanc’ and e2-9, *V. romanetii* C166-043, and *V. piasezkii* DVIT2027) were used in each round of disease evaluations to monitor the variation in the severity of the screen. The seedling replicates and control cultivars were randomized across the greenhouse and spaced 10 cm apart. The temperature of the greenhouse was set at 23–27 °C, lights were used to maintain a minimum 12 h day length if needed, and air humidity was elevated by spraying water on the floor. For inoculum, the C-isolate [35] was propagated on in vitro plated susceptible ‘Carignane’ leaves. On average approximately 70,000 conidia/ml in 0.1 % (v/v) Tween solution were used to infect each plant with a Perval Sprayer unit. Disease evaluations were carried out 4 weeks post inoculation by two people using a modified OIV-455 scale [36]: 0 - no symptoms, 1 - one or two small patches of PM on the entire plant, 2 - four to five patches of PM, 3 - many leaves have patches of PM, 4 - PM covers entire surface of many leaves on the same plant.

Microscopic evaluation of powdery mildew infections were made on in vitro cultured detached leaves for all breeding populations and for 10 accessions of *V. piasezkii* along with susceptible and resistant controls. Four fully expanded leaves from the third and fourth position on a shoot were collected, washed and plated as follows: rinse with distilled water, 2–3 min submergence into 0.3 % (w/v) sodium hypochlorite solution followed by four to five rinses with sterile distilled water, leaves were dried between sterile paper towels and petioles were trimmed before plating adaxial surface up onto 0.8 % agar in 100 x 15 mm Petri dishes. Leaves were inoculated using a settling tower procedure modified from Reifschneider and Boiteux [37] to obtain uniform and consistent powdery mildew infections with the C-isolate. A custom-made settling tower (50 ×50 ×120 cm) was attached to a vacuum system for 5 min followed by 10 min of conidia settling after breaking the vacuum. The average infection rate was 2.18 ± 1.5 conidia/mm^2^. Two people independently rated powdery mildew growth for all in vitro experiments at 14–15 days post inoculation (dpi) using a dissecting microscope (Leica EZ4 D) with the following scale: 0 - no hyphae, 1 - one or two conidia with hyphae, 2 - several conidia with secondary hyphae and establishment of micro colonies, 3 - mycelium on entire leaf surface, limited conidiophore, and 4 - mycelia coverage is extensive, reproduction is prolific, clearly visible with the naked eye. To obtain better visual observations, staining with Coomassie Brilliant Blue R-250 was carried out on detached leaves as described by Riaz et al. [35].

Phenotyping of the VpF1 progeny population was carried out using an Australian powdery mildew isolate (APC1) [34]. Inoculum was maintained on detached leaves of *V. vinifera* ‘Cabernet Sauvignon’ using an 8–10 day rotation and inoculated onto detached leaves of VpF1 progeny as previously described [38]. Scoring of the frequency of PCD induction in penetrated epidermal cells was carried out 2 dpi using trypan blue as previously described [14].

Molecular disease quantification on 247 genotypes was achieved with qPCR. For each genotype, infected leaves from the in vitro assay were collected after visual examination. The tissue was kept at − 20 °C until the DNA was extracted with a modified CTAB protocol with the addition of RNase treatment. The accumulated powdery mildew biomass was quantified by qPCR as described in Amrine et al. [39] with primer sequences designed for the *E. necator* elongation factor *EnEF1* gene (KHJ34692.1; [27]) along with *V. vinifera* actin-specific primers (Gene ID: 100232866; [40]). The reactions were carried out with SYBR Green Master mix as per the manufacturer’s instructions (Applied Biosystems 7500 Real-Time PCR System) using the following temperature profile: 2 min at 50 °C, 10 min at 95 °C, followed by 40 cycles of 15 s at 95 °C and 60s at 60 °C. DNA samples of three to four biological replicates of each genotype were randomized across reaction plates, and each plate had two replicates of reference *V. vinifera* and *E. necator* DNA samples. Infection coefficients from individual samples were calculated from linearized difference of C_T_ values with the formula: 2^-(*VvActin*CT-*EnEF1*CT)^.

A replicated subset of genotypes was also challenged with three additional powdery mildew isolates collected from different locations in California to determine if powdery mildew resistance is race-specific. Two of the tested isolates, Lodi and e1-101 were genetically distinct and grouped in a different clade based on their microsatellite profiles [27]. The isolate 11-373-J16 was collected from a susceptible seedling from the 11-373 population, which is maintained at UC Davis. All collected phenotypic data was analyzed using R 3.1.3 [41] and the Agricolae package [42].

### Genotyping and genetic map construction

Genomic DNA was extracted from young leaf tissue by a modified CTAB protocol [9]. A total of 277 progeny plants of the 11-373 population were used as a base mapping population to generate a framework genetic map. Five hundred and twenty SSR markers from previously published marker series were tested on a subset of eight samples including parents and progeny. The VMC and VMCNg marker series were developed by the *Vitis* Microsatellite Consortium (Agrogene, Moissy Crameyel, France), VVI series by Merdinoglu et al. [43], UDV series by Di Gaspero et al. [44], VChr series by Cipriani et al. [45], VVMS series by Thomas and Scott [46], SCU by Scott et al. [47], VVC by Decroocq et al. [48], VVMD by Bowers et al. [49, 50], and CTG, CF, AF primer sequences were derived from the EST-SSR database (University of California, Davis http://cgf.ucdavis.edu). The sequences of the primer pairs are available from the NCBI database (http://ncbi.nlm.nih.gov) and/or from the aforementioned references. To further saturate and refine the region for chromosome 9 and for chromosome 19, fourteen new SSR markers were developed (PN9 and PN19 series; Additional file 1: Table S1) utilizing the 12X genome sequence of PN40024 [51]. The genome sequence was screened with WebSat [52] for repetitive sequences and primers were designed with Primer3 software [53] using the following parameters: 35–60 % GC content, 22 bp length, and a calculated T_m_ of 60 °C [54].

Polymerase chain reactions of 10 μl volume were carried out with fluorescently-labeled forward primers using the following standardized thermocycling profile: 5 min at 95 °C followed by 35 cycles of 45 s at 95 °C, 45 s at 56 °C and 45 s 72 °C, followed by 10 min at 72 °C. Amplified products of up to five markers were combined depending on the amplicon size and fluorescent labels of the markers and run on an ABI 3500 capillary electrophoresis analyzer with GeneScan-500 Liz Size Standard (Life Technologies, Carlsbad, California, USA). Allele sizes were determined using GeneMapper 4.1 software (Applied Biosystem Co., Ltd., USA).

Markers were evaluated for Mendelian segregation ratios using *χ*^2^-tests and the parental and consensus genetic-linkage maps were created using JoinMap 4.1 [55]. Recombination frequencies were set between 0.25 and 0.05 to group the markers. The Kosambi mapping function was used to generate centimorgan (cM) distances [56]. In the interval regression mapping the independence LOD (logarithm of odd) was set to 5–8 with a one-step interval. Chromosome numbers and their orientation were derived from a consensus grape reference genetic map [57].

Additional *V. piasezkii* accessions were genotyped as described above with the following markers PN9-066.1, PN9-067, PN9-068, VMC4h6, VMC9a2.1, PN19-022 and VMC5h11. The allelic data was analyzed in DARwin6 [58] to generate a relationship tree with the unweighted neighbor-joining method employing 1000 bootstrap replications.

### Quantitative trait locus analyses

The quantitative trait locus (QTL) analysis for each trait was carried out using two different approaches with MapQTL 6.0 using both parental and consensus maps [59]. First, interval mapping (IM) analysis was carried out with a regression algorithm to detect possible QTLs on both parental maps. Automatic cofactor selection was carried out on five neighboring loci around the potential QTL with the *p* value set at 0.001 with 2000 iterations. In the next step, multiple QTL mapping (MQM) analysis was carried out for each phenotypic trait using the assigned cofactors from the previous step. To examine the effect of each locus independently, a subset of F1 11-373 progeny were selected based on local haplotypes for either *Ren6* or *Ren7* only and QTL analysis was carried out as described above. The genome wide, and combined significance LOD thresholds were calculated with 1000 permutations. The type-I error rate of 0.05 was used to identify significant LOD values.

### Additional pseudo-backcross breeding populations for key recombinant search

Four pseudo-backcross (pBC1) breeding populations were developed using resistant seedlings of 11-373 that inherited either *Ren6* or *Ren7* or both loci. In all four cases, PM resistant seedlings were used as the male parent and the susceptible *V. vinifera* ‘Malaga Rosada’ was used as the female parent. The populations 13-350, 13-351 (which segregated for *Ren6* only), 13-352 (both *Ren6* and *Ren7*) and 14353 (*Ren7* only) consisted of 396, 125, 133 and 256 seedlings, respectively. An additional 259 seedlings of the F1 11-373 population and all pBC1 populations were screened with markers flanking the *Ren6* and *Ren7* loci to identify potential recombinant plants. Disease evaluations were carried out on multiple replicates of all candidate recombinant plants as well as partial subsets of each population in the greenhouse and by the in vitro detached leaf assay. Inoculations and scoring was carried out using the experimental procedures described above.

### Gene annotation and identification of transposable elements

Based on the markers linked to the *Ren6* and *Ren7* loci, a 60 kb and 330 kb piece of corresponding genome sequence of PN40024 for each locus was scanned for the presence of transposable elements using CENSOR [60]. The gene annotations for the corresponding regions were obtained from *Gramene* [61] (12.1 assembly, V1 annotation). Both gene and transposable element annotations were overlaid and displayed using the software package Geneious v7.1.7. [62].

## Results

### Disease evaluations

The F1 11-373 seedling population was evaluated in multiple environments. Field evaluations for leaf and cane powdery mildew symptoms were carried out for two consecutive years (2013 and 2014) in addition to the greenhouse evaluations, in vitro assays, and qPCR evaluations. The Additional file 2: Table S2 provides the details on the number of seedlings tested in each year, minimum and maximum scores, means, and variances. Lower mean and variance was registered across all progeny for both leaf ($\overline{x}$=0.51, σ^2^ = 0.55) and cane ($\overline{x}$=0.23, σ^2^ = 0.29) evaluation in 2013 compared to 2014 ($\overline{x}$=1.29, σ^2^ = 3.09; $\overline{x}$=0.88, σ^2^ = 2.41 respectively for leaf and cane). The results of all methods used for disease evaluations were significantly correlated to each other (Table 1, *p* < 0.001). The pair wise correlations with the 2013 field scores and any other evaluation were lower (*R*^*2*^ ranging from 0.25 to 0.63). The highest correlation was observed between the visual scores from the in vitro assay in the controlled environment and the greenhouse assay (0.91). Likewise, high correlations were observed between the estimation of accumulated powdery mildew biomass by qPCR and phenotypic evaluations on greenhouse plants (0.77) as well as the in vitro assay (0.82).

**Table 1:** Correlation of average phenotypic scores across different disease evaluation screens

	Leaf 2013	Cane 2013	Leaf 2014	Cane 2014	Greenhouse	in vitro	qPCR
Leaf 2013	1.0	*-*	*-*	*-*	*-*	*-*	*-*
Cane 2013	0.632^a^	1.0	*-*	*-*	*-*	*-*	*-*
Leaf 2014	0.591	0.465	1.0	*-*	*-*	*-*	*-*
Cane 2014	0.633	0.590	0.827	1.0	*-*	*-*	*-*
Greenhouse	0.499	0.374	0.842	0.664	1.0	*-*	*-*
in vitro	0.409	0.283	0.786	0.617	0.910	1.0	*-*
qPCR^b^	−0.365	−0.245	−0.697	−0.546	−0.767	−0.818	1.0

^a^All *R* ^*2*^ values are significant (*p* < 0.001)

^b^The qPCR derived infection coefficients normalized with natural logarithm. They correlate inversely with the visual observations

In addition to testing the F1 11-373 population with the powdery mildew C-isolate, a subset of thirty-one F1 genotypes were also challenged with three additional powdery mildew isolates (Lodi, e1-101 and 11-373-J16 [27]) in the detached leaf assay. Analysis of variance detected no significant differences among the four powdery mildew isolates (Additional file 3: Table S5; *p* = 0.162).

### Marker analysis and genetic linkage maps

From a total of 520 markers, 268 and 264 were found to be polymorphic for the female and male parents, respectively. Two hundred and seven markers that were polymorphic for the resistant male parent and one marker for the female parent were applied to the base population of 277 seedlings. A total of 148 markers were fully informative, segregating for parents (ab × cd, ab × ac), and 59 were polymorphic for the male parent DVIT2027 only. The missing allelic information for the complete data set was 3.12 %. Of the 208 markers, 34 deviated from the expected Mendelian segregation (*p* < 0.05). All distorted markers are listed with *χ*^2^ values in Additional file 4: Table S3. Markers with significant deviation from Mendelian ratios were included on all maps if the order of the markers didn’t differ from previously reported maps. The flower phenotype was also evaluated for 180 seedlings that bloomed in 2014. Only pistillate and staminate flower phenotypes were observed and they segregated 1:1 (69:74, *χ*^2^ = 0.175 *p* = 0.6759).

Parental and consensus framework genetic maps were constructed with polymorphic marker data. The F2-35 parental map included 144 markers across 19 chromosomes covering 779.61 cM with an average marker distance of 5.41 cM. The DVIT2027 map included 207 markers across 19 chromosomes covering 1002.7 cM with an average marker distance of 5.35 cM. There were only seven gaps that were bigger than 20 cM (Additional file 5: Figure S1). The consensus genetic map was 1005.4 cM with an average marker distance of 5.31 cM. Eight newly designed SSR makers (PN9 series) were mapped to their respective location in both parental and consensus genetic maps. In all three maps the order of the markers was consistent and comparable to known reference maps. The parental and consensus framework maps represented complete coverage of the genome based on the markers that are common to other published maps. The summary statistics of both parental and consensus maps are presented in Table 2. Sixteen of thirty-four markers with significant segregation distortion mapped to chromosome 1 (Additional file 4: Table S3). The flower sex phenotype, as a qualitative marker, mapped to chromosome 2 (data not shown), the same genomic region as reported in previous studies [63–65].

**Table 2:** Summary of the consensus and the two parental genetic framework maps

Chromosome	Consensus Map			DVIT2027 Map			F2-35 Map		
	No. Markers	cM	Average Distance (cM)	No. Markers	cM	Average Distance (cM)	No. Markers	cM	Average Distance (cM)
1	16	65.89	4.12	16	63.02	3.94	11	74.59	6.78
2	10	35.76	3.58	10	33.15	3.31	3	14.82	4.94
3	6	47.77	7.96	6	47.14	7.86	5	12.81	2.56
4	10	70.82	7.08	10	76.05	7.60	9	46.74	5.19
5	12	28.20	2.35	12	24.10	2.01	9	34.62	3.85
6	13	57.66	4.44	13	58.60	4.51	10	53.75	5.37
7	4	39.97	9.99	4	39.48	9.87	3	12.92	4.31
8	9	48.79	5.42	9	49.51	5.50	5	40.90	8.18
9	18	57.73	3.21	18	53.06	2.95	13	61.64	4.74
10	7	48.13	6.88	7	47.16	6.74	4	26.38	6.59
11	8	49.83	6.23	8	51.21	6.40	5	46.04	9.21
12	8	50.91	6.36	8	46.17	5.77	5	34.63	6.93
13	12	55.94	4.66	12	56.98	4.75	8	42.19	5.27
14	15	63.27	4.22	15	62.16	4.14	9	65.63	7.29
15	4	24.68	6.17	4	19.44	4.86	4	30.10	7.52
16	11	61.56	5.60	11	73.79	6.71	6	15.18	2.53
17	9	50.86	5.65	9	52.71	5.86	6	35.84	5.97
18	14	89.72	6.41	13	92.32	7.10	12	76.19	6.35
19	22	57.94	2.63	22	56.67	2.58	17	54.65	3.21
Total	208	1005.42	4.83	207	1002.71	4.84	144	779.61	5.41

### QTL-analysis

The QTL analysis was carried out by Interval mapping (IM) and Multiple-QTL Mapping (MQM) using both parental and consensus maps. Significant QTLs were detected on the consensus and DVIT2027 parental maps, but not on the F2-35 map. From hereon we present the QTL results of the male parental map only, since the resistance is derived exclusively from *V. piasezkii* DVIT2027.

**Fig. 2:**
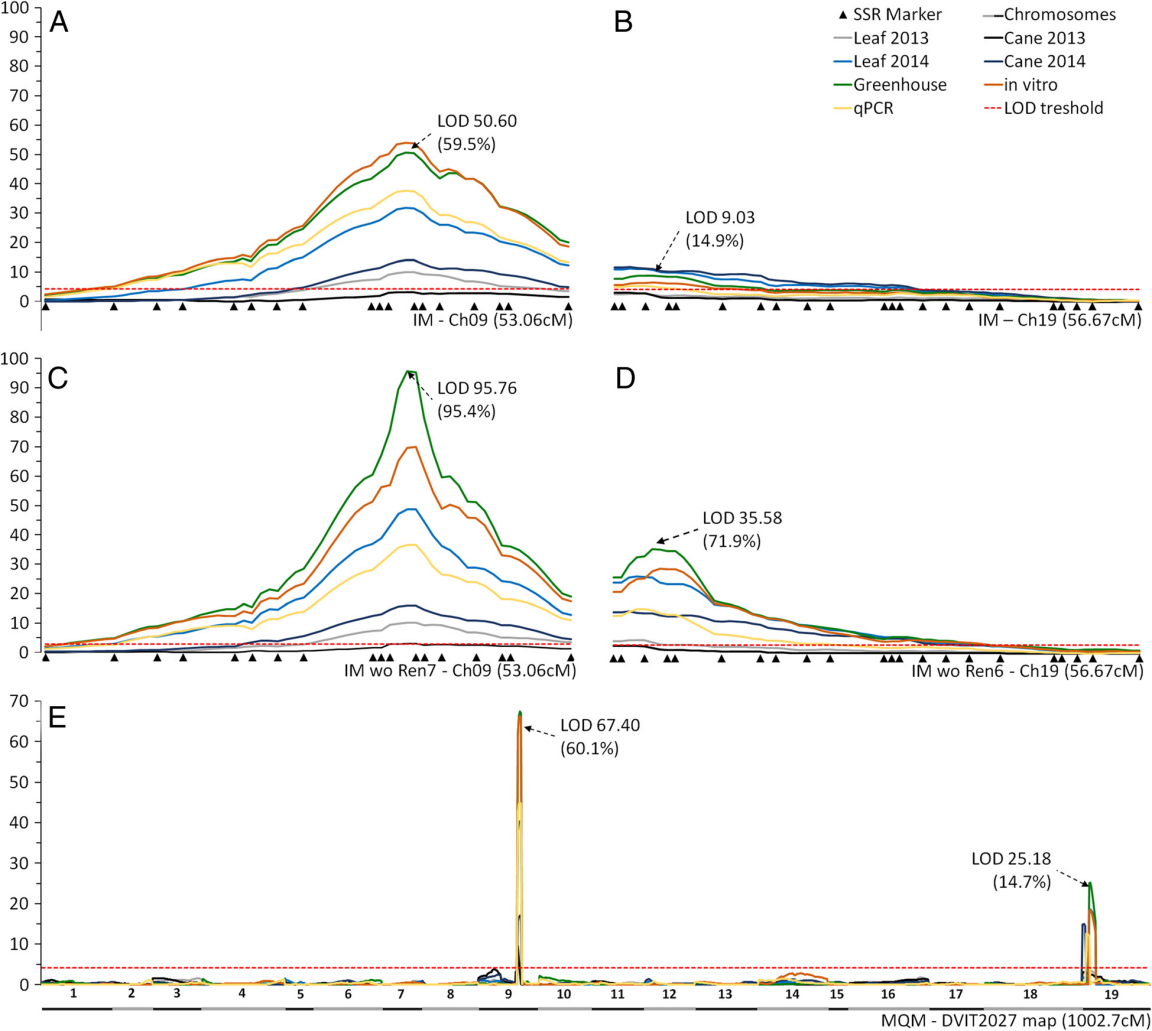
Summary of powdery mildew susceptibility of the four genotypic classes within the F1 population. Susceptibility was assessed on leaves and canes of field-grown vines in 2013 using natural powdery mildew inoculations, as well as artificial inoculations in the field during 2014 of greenhouse plants and detached leaves in vitro. A 6-point rating scale, 0 (no visible symptoms) to 5 (powdery mildew covers all tissue) was used to determine powdery mildew susceptibility for field evaluations in 2013 and 2014. Powdery mildew susceptibility was rated using a 5-point scale, 0 (no symptoms) to 4 (powdery mildew covers majority of the leaves) in the greenhouse and in vitro assays. Significant differences detected with Tukey’s test are indicated with different letters. The letter ‘n’ denotes the number of genotypes used for analysis in each of the disease evaluation methods. The *E. necator* biomass was measured by qPCR, plotted infection coefficients correspond to natural logarithm-transformed 2^-ΔCT^ values. The higher values indicate less biomass accumulation

The IM analysis identified two resistance loci, the first on chromosome 9 (*Ren6*) and the second on chromosome 19 (*Ren7*). SSR marker PN9-057 and PN9-068 flanked the LOD peak for the *Ren6* locus on chromosome 9. The position of the *Ren6* locus did not change with the method of disease evaluation (Fig. 2a; Additional file 6: Table S4). However, the phenotypic variation explained by the *Ren6* locus varied with the method of disease evaluation. The maximum variation explained (61.9 %) was with the controlled in vitro screen method with a LOD 54.3 (Additional file 6: Table S4). The above-mentioned flanking markers for the *Ren6* locus were used for all subsequent screens for recombinants in additional seedlings of the F1 and pBC1 populations. The IM analysis identified VVIp17.1 and VMC9a2.1 as flanking markers for the *Ren7* locus for the 2013 and 2014 field leaf and cane disease evaluations. However, for the greenhouse, in vitro and qPCR assay, the flanking markers were VMC9a2.1 and VMC5h11 (Fig. 2b; Additional file 6: Table S4). The *Ren7* locus explained 19 % phenotypic variation with a LOD 11.92 for the cane evaluation from 2014. All three SSR markers (VVIp17.1, VMC9a2.1 and VMC5h11) were used to identify recombinants in additional F1 and pBC1 populations.

Multiple-QTL mapping analysis confirmed the two previously identified loci with the IM approach (Fig. 2e). The automatic cofactor selection procedure identified the PN9-068 marker as a cofactor for all disease evaluation approaches except for the 2013 field data for the *Ren6* locus. With the PN9-068 marker as a cofactor, phenotypic variation explained by the *Ren6* locus varied across the method of disease evaluation. A maximum of 62 % variation was also observed for in vitro analysis (Additional file 6: Table S4) with LOD 66.28. For the *Ren7* locus on chromosome 19, the VVIu09 marker was selected as a cofactor for the greenhouse and in vitro assay and VMC5h11 was used for the qPCR analysis. Both markers are closely linked and are only 0.9 cM apart on the map (Additional file 5: Figure S1). A maximum of 18.1 % variation was observed for the 2014 cane screen with LOD 14.55. The detailed results of IM and MQM are presented in Additional file 6: Table S4. The alleles of SSR markers that are linked to the *Ren6* and *Ren7* loci are presented in Tables 3 and 4.

**Table 3:** Allelic profiles of *Ren6* flanking markers on chromosome 9

ID	PN9-057 ^a^	PN9-063	PN9-066.1	*Ren6*	PN9-067	PN9-067.2	PN9-068	VMC4h4.1	in vitro PM	*Ren7*
F2-35	185/187	170/188	390/434	-	284/290	303/303	null/null	247/251	4.00	-
Malaga Rosada	185/185	188/null	387/434	-	284/292	303/303	180/null	211/251	4.00	-
DVIT2027	**190**/204	166/**174**	**402**/408	+	281/**288**	314/**320**	171/**174**	**178**/229	0.00	+
11373-473	-	+	+	+	+	+	+	+	0.50	-
11373-483	-	+	+	+	+	+	+	+	0.00	-
11373-014	-	-	+	+	+	+	+	+	0.25	-
11373-094	-	-	+	+	+	+	+	+	0.50	+
11373-128	-	-	+	+	+	+	+	+	0.00	-
11373-390	-	-	+	+	+	+	+	+	0.00	-
11373-148	-	-	-	-	-	-	+	+	1.75	+
11373-245	-	-	-	-	-	-	+	+	1.58	+
11373-276	-	-	-	-	-	-	-	+	4.00	-
11373-497	-	-	-	-	-	-	-	-	2.38	+
13350-357	+	+	+	+	+	-	-	-	0.00	-
13351-057	+	+	+	-	-	-	-	-	4.00	-
13350-055	+	+	-	-	-	-	-	-	4.00	-
13351-020	+	+	-	-	-	-	-	-	4.00	-

^a^Underlined marker names are included in the framework map

**Table 4:** Allelic profiles of *Ren7* flanking markers on chromosome 19

ID	VVin74	VVip17.1	PN19-018	VMC9a2.1	PN19-022	*Ren7*	VMC5H11	VVIu09	in vitro PM	*Ren6*
F2-35	278/278	77/77	null/null	163/163	274/null	-	195/198	95/97	4.00	-
Malaga Rosada	278/288	77/87	null/180	163/163	261/274	-	181/195	95/97	4.00	-
DVIT2027	278/**280** ^a^	77/**79**	null/**187**	**163**/165	268/**268**	+	175/**200**	**99**/104	0.00	+
13352-012	-	-	+	ud^b^	+	+	+	+	0.50	+
11373-497	-	-	-	+	ud	+	+	+	2.38	-
14353-026	-	-	-	ud	+	+	+	+	0.81	-
14353-028	-	-	-	ud	+	+	+	+	0.83	-
11373-186	-	-	-	-	ud	+	+	+	2.25	-
11373-415	-	-	-	-	ud	ud	+	+	0.00	+
13352-025	-	-	-	ud	-	-	+	+	4.00	-
11373-471	+	+	+	+	ud	+	-	-	1.88	-
14353-213	+	+	+	ud	+	+	-	-	2.00	-
11373-001	+	+	+	+	ud	ud	-	-	0.00	+
11373-008	+	+	+	+	ud	-	-	-	3.75	-
11373-075	+	+	+	+	ud	ud	-	-	0.13	+
11373-088	+	+	+	+	ud	ud	-	-	0.00	+
11373-150	+	+	+	+	ud	-	-	-	3.50	-
13352-004	+	+	+	ud	-	-	-	-	4.00	-
13352-015	+	+	+	ud	-	-	-	-	4.00	-
14353-126	+	+	+	ud	-	-	-	-	3.66	-
14353-151	+	+	+	ud	-	-	-	-	4.00	-
14353-082	+	+	-	ud	-	-	-	-	4.00	-
14353-086	+	+	-	ud	-	-	-	-	3.25	-
14353-214	+	+	-	ud	-	-	-	-	4.00	-
14353-223	+	+	-	ud	-	-	-	-	4.00	-

^a^Alleles shown in bold represent the resistant haplotypes

^b^Undetermined is shown as ‘ud’

To study the effect of each locus independently, F1 progeny were divided into groups based on the presence of *Ren6* and *Ren7* haplotypes. All genotypes with the *Ren6* linked allele (PN9-068, 174 bp) were removed from the datasets, and IM analysis was applied to the remaining genotypes that theoretically only segregated for *Ren7*. The IM analysis in the absence of *Ren6* boosted the impact of the *Ren7* locus to 71.9 % explained variation at LOD 35.58 with the greenhouse screen data. The IM analysis was also performed inversely, with genotypes containing the *Ren7* linked allele (VMC9a2.1, 163 bp) removed from the genotype file. The *Ren6* locus explained as much as 95.4 % of the phenotypic variation (LOD 95.76) in the absence of *Ren7* (Fig. 2c–d; Additional file 6: Table S4).

To further demonstrate that there were no other genetic factors contributing to powdery mildew resistance, the dataset was reanalyzed following removal of all genotypes with alleles linked to *Ren6* or *Ren7*. Interval mapping on this data set did not reveal any other significant QTLs.

### Independent assortment of *Ren6* and *Ren7* loci in terms of powdery mildew resistance

**Fig. 3:**
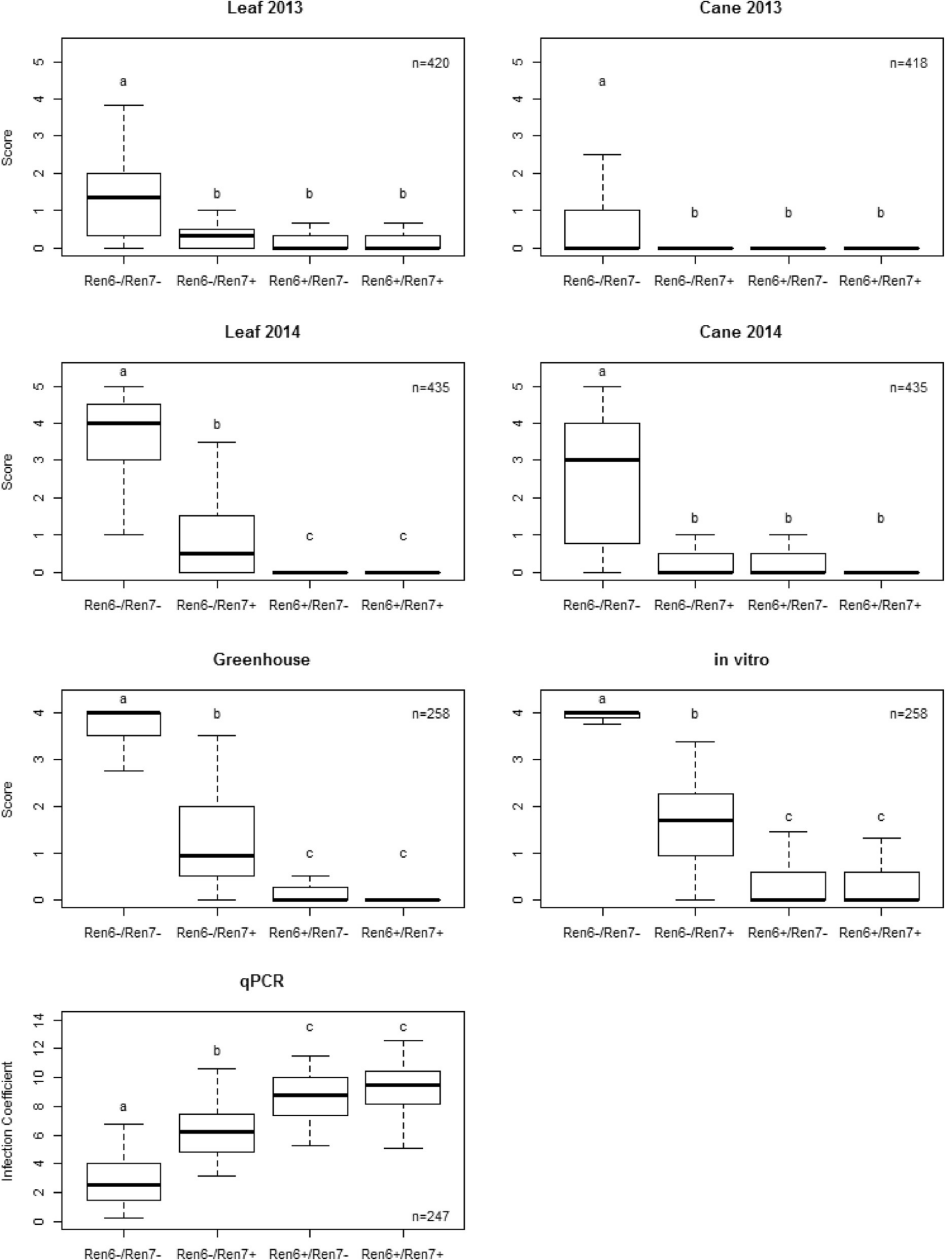
Comparative development of powdery mildew on in vitro leaves of genotypes containing different combinations of R loci introgressed from *Vitis piasezkii.* Detached leaves were inoculated with powdery mildew using a settling tower, harvested 5 dpi and stained with Coomassie Brilliant blue to visualize the development of fungal structures on the leaf surface. (**a**) *Ren6* ^−^/*Ren7* ^−^, (**b**) *Ren6* ^−^/*Ren7* ^+^, (**c**) *Ren6* ^+^/*Ren7* ^−^, and (**d**) *Ren6* ^+^/*Ren7* ^+^ genotypes. The *brown* cells beneath the appressoria of germinated fungal spores are the result of a hypersensitive response induced by the R loci. Scale bars represent 50 μm

**Fig. 4:**
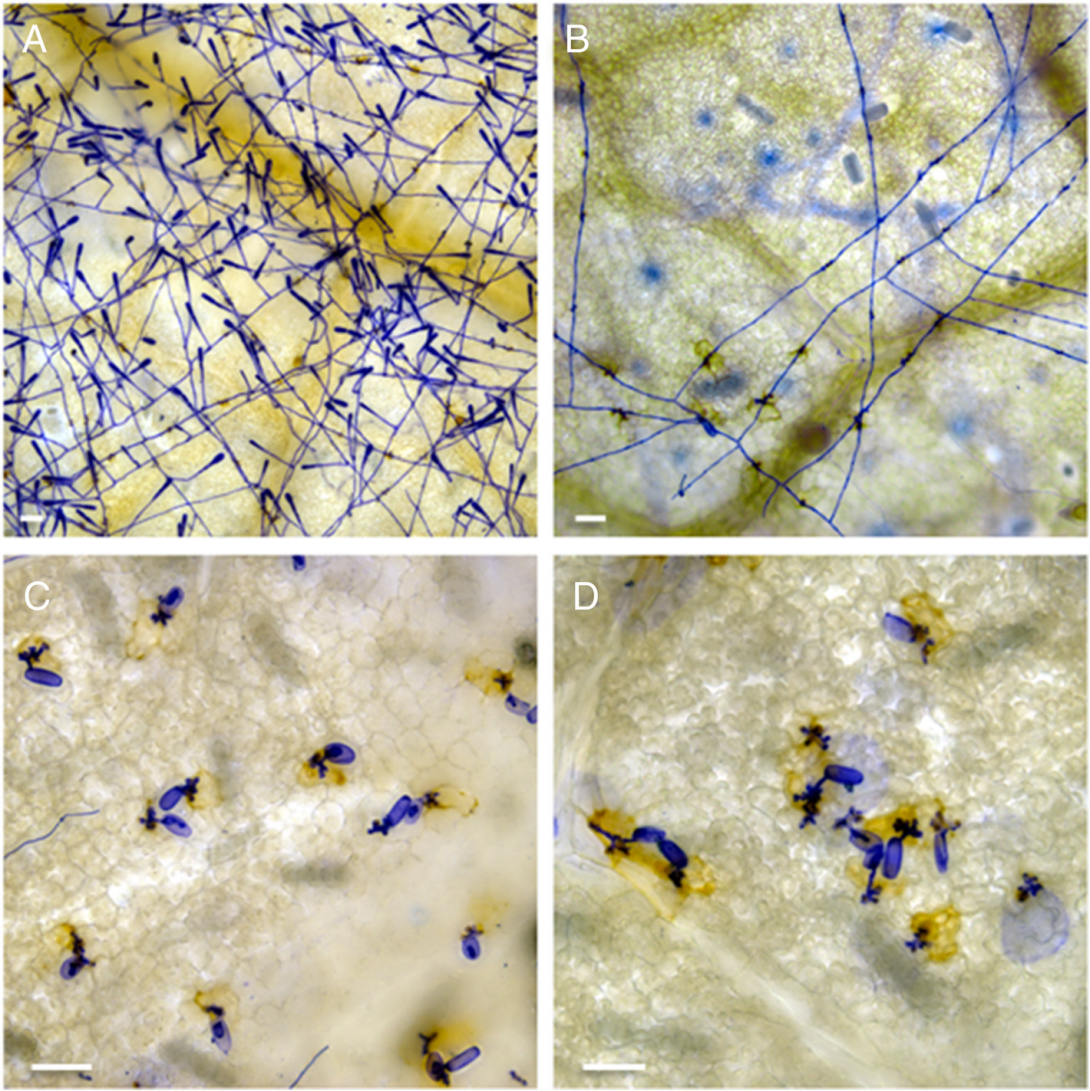
Comparison of PCD (programmed cell death) induction kinetics in *Ren6* and *Ren7* genotypes. All data and micrographs were collected 2 dpi and at least 100 germinated spores were scored following trypan *blue* staining for estimation of PCD. (**a**) Relative levels of PCD in powdery mildew-penetrated epidermal cells of an F1 microvine population segregating for *Ren6* and *Ren7*. Each data point is the mean ± SE of at least two biological replicates. (**b**) Proportion of penetrated epidermal cells that show either effective PCD (no secondary hyphae), ineffective PCD (secondary hyphae produced) or no PCD following powdery mildew penetration in four individual VpF1 lines shown in (**a**). For comparison, two additional microvine lines were included – a susceptible line lacking any R genes and a resistant line containing the *Run1* locus. Results are shown from one experiment, but the experiment was repeated twice with the same results. (**c**–**f**) Micrographs showing examples of effective PCD (**c**, **d**) and non-effective PCD (**e**, **f**). Epidermal cells that have undergone PCD, as shown by the uptake of trypan *blue*, are indicated with an asterisk while secondary hyphae are indicated by *white arrows*

The two newly identified loci, *Ren6* on chromosome 9 and *Ren7* on chromosome 19, segregate independently of each other and generated four classes of genotypes (*Ren6*^+^/*Ren7*^+^, *Ren6*^+^/*Ren7*^−^, *Ren6*^−^/*Ren7*^+^ and *Ren6*^−^/*Ren7*^−^). Theoretically, we should expect equal ratios of four phenotypic classes in the F1 progeny since the female parent is susceptible to powdery mildew, lacks both loci and does not contribute any minor genes for resistance. The ratio of the four genotypes was 63:73:61:79 (*χ*^2^ = 3.245 *p* = 0.3553) confirming that both loci segregate independently of each other and followed Mendel’s second law of inheritance. A Tukey’s test significantly separated the phenotypic scores of susceptible progeny (*Ren6*^−^/*Ren7*^−^) from the genotypes that have either *Ren6* or *Ren7* or both loci. Significant phenotypic differences were detected between *Ren6*^*+*^*/Ren7*^*+*^ or *Ren6*^*+*^*/Ren7*^*−*^ and the *Ren6*^*−*^*/Ren7*^*+*^ genotypes, in the 2014 field leaf scores, greenhouse, in vitro, and qPCR evaluations (Fig. 3). These differences were also clearly illustrated by the powdery mildew development observed on leaves from the different genotypes in the in vitro assay (Fig. 4). Inoculated leaves were harvested 5 dpi and stained with Coomassie Brilliant blue to visualize the development of fungal structures on the leaf surface. Genotypes lacking both *Ren6* and *Ren7* (Fig. 4a) showed extensive hyphal growth and conidiophore development after 5 dpi. On *Ren6*^−^/*Ren7*^+^ genotypes (Fig. 4b), secondary hyphae were clearly visible on the leaf surface but the density was markedly reduced compared to the fully susceptible *Ren6*^−^/*Ren7*^−^ genotypes. In a very few cases, minor amounts of conidiophore formation was observed on some *Ren6*^−^/*Ren7*^+^ leaves at 14 dpi in the in vitro assay represented by the error bars on Fig. 3. Powdery mildew development on leaves of genotypes containing *Ren6*^+^/*Ren7*^−^ and *Ren6*^+^/*Ren7*^*+*^ was very similar (Fig. 3, Fig. 4c–d) with little or no secondary hyphae development. The disease symptoms on canes were not significantly different between *Ren6*^*+*^*/Ren7*^*+*^ or *Ren6*^*+*^*/Ren7*^*−*^ and Ren6^−^/Ren7^+^ for both years in the field. There was no separation of *Ren6*^+^/*Ren7*^+^ and *Ren6*^+^/*Ren7*^−^ from each other with the 2013 and 2014 leaf scores, greenhouse screen, in vitro screen and qPCR results confirming the strong influence of the *Ren6* locus on the phenotype (Fig. 3).

### Characterization of the *Ren6* and *Ren7* resistance response

There is clear evidence from Fig. 4 of a hypersensitive response (HR) to powdery mildew inoculation in genotypes containing either *Ren7* (Fig. 4b) or *Ren6* (Fig. 4c). In the case of *Ren7* this was mainly associated with epidermal cells penetrated by appressoria on developing secondary hyphae, whereas in *Ren6* genotypes the HR appeared to be more pronounced and was associated with appressoria of germinated spores. This HR is most likely the result of the penetrated epidermal cells undergoing PCD following recognition of specific avirulence effectors secreted by the invading powdery mildew pathogen [66]. However, the strength or speed of the PCD response and its effectiveness in restricting hyphal development appears to differ significantly between *Ren6* and *Ren7*.

To further investigate these differences and to enable us to compare the PCD response mediated by the two *R* loci from *V. piasezkii* to the previously characterized *Run1* locus*,* all three *R* loci were introduced into the same genetic background by crossing with the powdery mildew-susceptible *V. vinifera* ‘Pinot Meunier’ mutant picovine [33] and disease phenotypes were observed in response to a grapevine powdery mildew isolate from Australia.

**Fig. 5:**
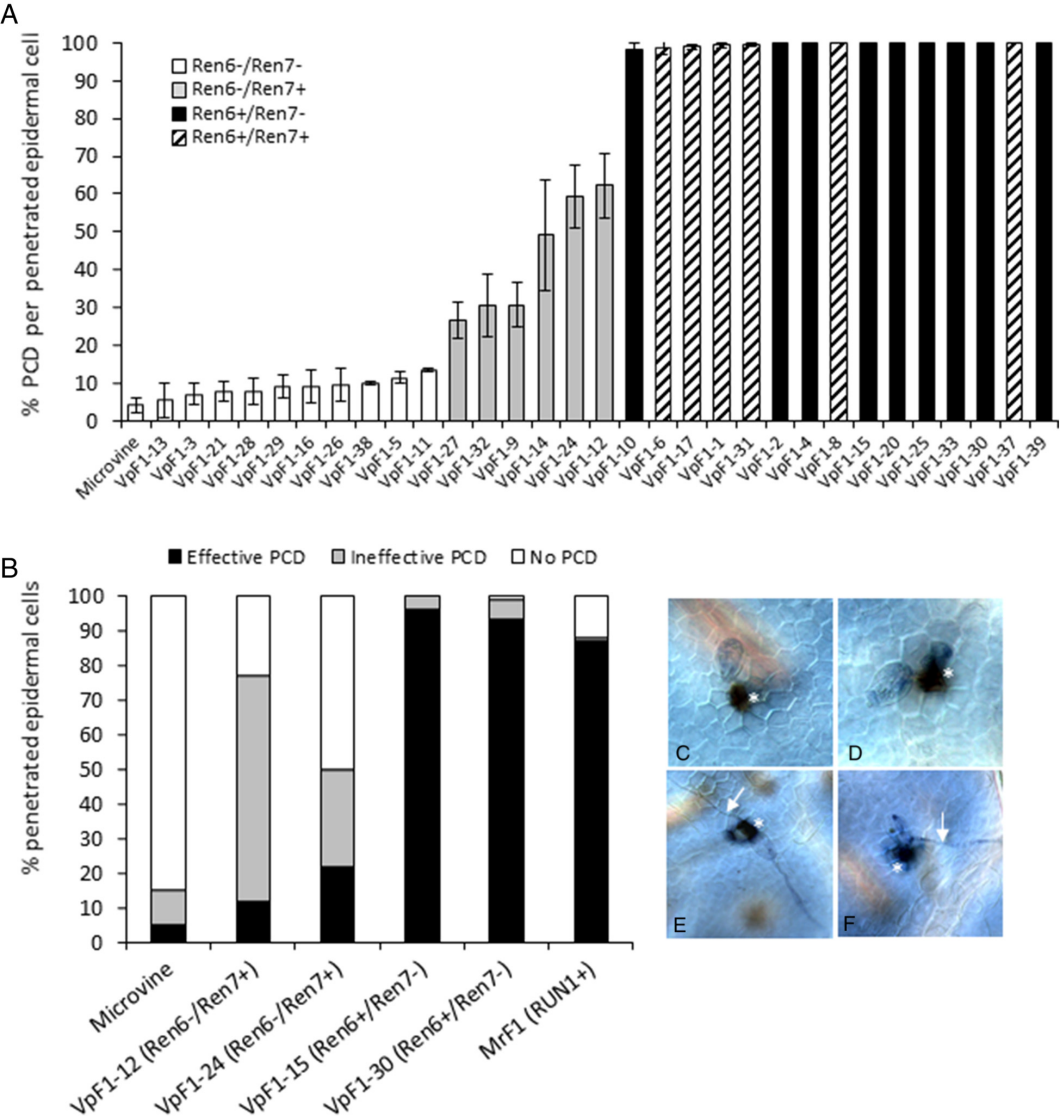
Origin and genetic characterization of *Vitis piasezkii* accessions used in this study. **a** Nine accessions including DVIT2027 were collected in the Shennongjia Forest, Hubei Province, China in 1980. The map of China was drawn using R 3.1.3 [41], package maps [76]; **b** Unweighted neighbor joining tree derived from local haplotypes; **c** Local haplotype of *V. piasezkii* accessions and results of in vitro powdery mildew resistance evaluations. Collection site # within the Shennongjia Forest as cited in Bartholomew et al. [30]. The genetic marker data used to construct the dendrogram can be found at (http://purl.org/phylo/treebase/phylows/study/TB2:S19503)

A small F1 population (VpF1) of 31 progeny was generated from the cross of DVIT2027 with picovine line 06C008V0003. The *Ren6* marker PN9-067 and the *Ren7* markers VMC9a2.1 and VMC5h11 were found to be informative in this cross and used to genotype the progeny. Percent induction of PCD in penetrated epidermal cells was measured 2 dpi using the vital stain trypan blue that is only taken up by dead plant cells [14]. Figure 5a shows that the powdery mildew resistance response mediated by both *Ren6* and *Ren7* involves the induction of PCD in penetrated epidermal cells. This observation confirms that these two *R* loci from *V. piasezkii* are able to recognize powdery mildew isolates from both California and Australia. It also indicates that the PCD-based resistance response mediated by *Ren6* is stronger or more rapid than that mediated by *Ren7*. This is confirmed by the results of a separate study that compared powdery mildew induced PCD induction in selected lines of *Ren6*^−^/*Ren7*^+^, *Ren6*^+^/*Ren7*^−^ and *Run1* F1 progeny in the same genetic background (Fig. 5b). The results are presented in terms of the percentage of fungal penetrated epidermal cells that have either undergone either effective PCD (no secondary hyphae formation), ineffective PCD (secondary hyphae formation still occurs) or no PCD. The VpF1 progeny containing *Ren6* displayed a very high incidence of effective PCD (i.e. >93 %) in penetrated epidermal cells leading to complete suppression of secondary hyphae formation. In contrast, VpF1 progeny containing *Ren7* displayed much lower levels of effective PCD (<22 %) in penetrated cells and much higher levels of ineffective PCD (28–65 %), which resulted in much greater levels of secondary hyphal growth. Based on these results, *Ren6* appears to mediate a more rapid/stronger PCD response to powdery mildew infection than *Run1*, while the *Ren7* mediated response is slower/weaker than *Run1*.

### Search for additional key recombinants

The screening of additional genotypes of the F1 population 11-373 and the four derived pBC1 populations with markers linked to *Ren6* and *Ren7* loci allowed the identification of additional recombinant genotypes. In the 2.2 cM genetic window of the *Ren6* locus (between PN9-057 and PN9-068) 13 recombination events were identified from 1169 seedlings. To further refine the 2.3 cM wide genetic window of the *Ren7* locus, 917 seedlings were evaluated with flanking markers. Nine recombinants were found in the F1 population (*n* = 536), five of them lacking the *Ren6* locus. In addition, two pBC1 populations (*n* = 386) were screened within a wider genetic window because of the homozygosity of the VMC9a2.1 marker in the resistant pBC1 parents. Thirteen recombinants were identified; 12 of them did not possess *Ren6*. The haplotype and phenotype of recombinants for both loci are summarized in Tables 3 and 4.

In the refined genetic map based on the additional recombinant genotypes, the *Ren6* locus resides between markers PN9-066.1 and PN9-067 (Tables 3 and 4). The physical distance between these two markers in the PN20024 genome sequence is 22 kb. The refined genetic map of the *Ren7* locus consisted of two new microsatellite markers (PN19 series, Additional file 1: Table S1). The *Ren7* locus resides between PN19-022 and VMC5h11 and the corresponding physical distance between these two markers in the PN20024 genome sequence is 330 kb (Tables 3 and 4).

### Genotyping and phenotyping of additional *V. piasezkii* accessions

The detached leaf in vitro assay was carried out on nine additional accessions of *V. piasezkii* maintained at the USDA National Clonal Germplasm Repository, Davis, California. Eight of these accessions were collected from the Shennongjia Forestry District, and one from an undetermined location in China (Fig. 1a). SSR markers linked to the *Ren6* and *Ren7* loci were used to genotype these accessions to identify other similar haplotypes based on their genotypic and phenotypic profiles. The results of genotyping and phenotyping of these accessions are presented in Fig. 1c. The in vitro test was carried out with the C-isolate only and identified seven accessions that exhibited varying levels of resistance to PM. Three accessions (DVIT2026, DVIT2028, and DVIT2032) were resistant to PM in the detached leaf assay and were positive for the SSR marker allele(s) that linked the *Ren6* locus in DVIT2027 (Fig. 1c). Interestingly, DVIT2026 had both the *Ren6* and *Ren7* linked SSR marker alleles and showed complete immunity to PM in the in vitro assay. Five accessions had SSR marker allele(s) that were linked to the *Ren7* locus in DVIT2027 (Fig. 1c). Unweighted neighbor joining analysis placed DVIT2026 and DVIT2027 in the same clade indicating that they are closely related to each other. Two other *Ren6* like haplotypes (DVIT2028 and DVIT2032) were in different clades; both of them were collected from different sites in the Shennongjia Forestry District (Fig. 1b). The accessions similar to the *Ren7* haplotype based on the linked markers showed variation in PM infection with symptoms ranging from 1.33 to 2.13 (Fig. 1c).

## Discussion

### *Vitis piasezkii* has two unique loci to restrict powdery mildew infection

In this study we (i) explored powdery mildew resistance in ten accessions of the Chinese species, *V. piasezkii*, (ii) developed F1 and pBC1 breeding populations with a single resistance source and (iii) identified two loci *Ren6* and *Ren7* on different chromosomes, chromosome 9 and chromosome 19, respectively. Powdery mildew resistance has not been found to be associated with these chromosomes in previously published studies [9–12, 14, 19, 20, 26]. The identification of *Ren6* and *Ren7* loci was supported with disease evaluation data obtained from multiple environments. Field evaluations for both leaf and cane symptoms were carried out for two consecutive years without fungicide applications, and this data was confirmed by assays in the greenhouse, in vitro on detached leaves, and with qPCR assays. In general, field evaluation results may vary from year to year depending on the inoculum pressure which is strongly influenced by the weather, population biology and strain composition for any given year within a vineyard [67]. In agreement with previous reports, we observed that the maturity of the plants plays a role in the variation of disease severity [68]. We also observed variation in the field evaluation results between the 2 years of data collection that was reflected in the different values of phenotypic variation explained by both loci in QTL analysis (Fig. 2; Additional file 2: Table S2 and Additional file 6: Table S4). Nonetheless, we identified the *Ren6* and *Ren7* loci with significant LOD scores in the same genomic regions, independently of the type of phenotypic data used for the analysis. Identification of two *R* loci for the same pathogen that segregate independently of each other is novel for grape, but has been reported for other crops. For example, two loci were identified for potato virus X (PVX) resistance in potato [69], brown planthopper (*Nilaparvata lugens* Stål.) resistance in rice [70], clubroot (*Plasmodiophora brassicae* Woronin.) resistance in *Brassica oleracea* and in *B. rapa* [71, 72].

We observed four genotypic classes with a 1:1:1:1 segregation in the progeny. However, the phenotypic scores statistically divided into three groups for the leaf data from the field in 2014, the controlled greenhouse assay, in vitro scoring, and qPCR results. This was a result of the difficulty in separating the genotypes that carry *Ren6*^+^/*Ren7*^+^ and *Ren6*^+^/*Ren7*^*−*^ due to the strong effect of the *Ren6* locus on the phenotype. Interestingly, for the cane evaluations for both years, the *Ren6*^−^/*Ren7*^+^ phenotype was also not distinguishable from the two resistant genotypic classes of *Ren6*^+^/*Ren7*^+^ and *Ren6*^+^/*Ren7*^*−*^ (Fig. 3). These results indicate that under normal field conditions, the *Ren7* locus could provide effective resistance to cane tissue against the powdery mildew infection. Variation in the level of the powdery mildew resistance between different tissue types was observed in an earlier study [9]. However, no information is available about the underlying factors that might contribute to this variation.

### *Ren6* and *Ren7* confer resistance at the post-penetration phase

There is a significant amount of diversity and variation for powdery mildew disease resistance within the grape genome as demonstrated by the identification of numerous *R* loci from a wide range of wild *Vitis* species. Prior to this work, seven *R* loci had been mapped in different *Vitis* species from North America as well as Central Asia and China (see review by Qiu et al. [66]). Among all of the loci mapped, the exact position and identity of the gene conferring the resistance at the locus has only been resolved for the *Run1* locus [34, 66]. The *Run1* locus was found to comprise a family of seven putative Toll/interleukin-1 receptor (TIR)-NB-LRR-type *R* genes, one of which designated *MrRUN1* was found to confer strong resistance in transformed *V. vinifera* cultivars that were otherwise susceptible to the PM infection [34]. The *MrRUN1* gene confers resistance via the rapid induction of PCD in penetrated epidermal cells, restricting the availability of nutrients for further growth and development of the fungus. Other powdery mildew *R* loci such as *Run2*, *Ren1* and *Ren2* exhibit a lower frequency of PCD of penetrated cells compared to *Run1* allowing more extensive secondary hyphal development [14]. In the case of *Ren1*, the fungus is able to obtain sufficient nutrition to complete its life cycle, although the level of sporulation is approximately 10-fold lower than that observed on susceptible genotypes [19].

It is clear that, like *Run1*, both the *Ren6* and *Ren7* loci from *V. piasezkii* confer resistance to powdery mildew through the induction of PCD following fungal penetration (Fig. 5). However, the speed and/or strength of PCD induction vary markedly between these two loci. In the presence of *Ren6*, PCD induction is extremely rapid with 92–95 % of epidermal cells displaying effective PCD i.e. no development of secondary hyphae, after 2 dpi (Fig. 5). The *Ren6* resistance response is even stronger than that mediated by the *Run1* locus in the same genetic background (Fig. 5). In contrast, the resistance response of *Ren7* genotypes is much slower than *Ren6* resulting in a high percentage of penetrated epidermal cells in which either no PCD is observed or the PCD induction can be considered ineffective because the fungus is still able to produce a secondary hyphae (Fig. 4, 5). What is responsible for the differences in the speed or strength of the post-penetration PCD induction mediated by each of these different R proteins? One possibility is that each of these proteins recognizes different core effectors secreted by *E. necator* and that these effectors are secreted at different stages during the infection process or at markedly different levels. A second possible explanation is that the differences in speed or strength of PCD induction is a reflection of differences in the steady state level of the R protein within the grape leaf epidermal cells. A good demonstration of the influence of R protein levels on the kinetics of the resistance response comes from work on the barley powdery mildew resistance gene MLA12. Shen et al. [73] were able to convert the slow-acting resistance response of MLA12 into a rapid response by over-expression of MLA12 in barley cells with a strong ubiquitin promoter, suggesting that cellular amounts of MLA12, or protein complexes containing MLA12, are rate limiting for the onset or speed of the resistance response.

### Presence of PM resistance in Central Asia and China

*Vitis piasezkii* is the second Chinese species known to confer strong resistance to powdery mildew for which the *R* locus has been mapped. Powdery mildew resistance was previously mapped to the *Ren4* locus in *V. romanetii* [9, 26]. Many Central Asian cultivated and wild accessions of *V. vinifera* spp. *sylvestris*, the progenitor of the cultivated *V. vinifera* spp. *sativa*, were also identified to carry partial resistance to the PM [19–21, 39]. The presence of strong resistance to powdery mildew in Asian *Vitis* species appears to be at odds with the current theory regarding the co-evolution of *E. necator* on wild North American grapevines and its subsequent introduction into Europe and to the rest of the world in the mid-nineteenth century [23]. Such a time frame would clearly have been insufficient time, in evolutionary terms, for resistance to develop in the Asian *Vitis* species [21].

The presence of two different R loci to avoid powdery mildew infection is another intriguing aspect that poses more questions. Did these loci evolve independently of each other, or was one derived from the other? The possible answer to this question lies in the comparative sequence analysis of the genomic regions carrying these loci to other sequenced grape genomes. No significant disease resistance-related candidate genes were identified in 22 kb and an expanded 60 kb corresponding genomic region for the *Ren6* locus and a 330 kb region for the *Ren7* locus in the susceptible *V. vinifera* PN40024 (12X.1) reference genome sequence (Additional file 7: Figure S2). It is likely that genetic and physical distances between the Chinese *V. piasezkii* and the European PN40024 do not correlate with each other and prevent accurate comparisons of the two genomes (Tables 3 and 4). The other hypothesis is that *V. piasezkii* possesses unique genes that are not present in this region of the PN40024 genome sequence. The corresponding regions for both loci in the PN40024 genome sequence had large clusters of retro- and DNA-transposable elements, which are common around and between clusters of disease resistance genes [20]. Physical maps for the *Ren6* and *Ren7* loci will allow direct comparisons to other susceptible genomes and help examine gene structure for these loci.

The other *V. piasezkii* accessions acquired from the Shennongjia Forestry District contained either *Ren6* or *Ren7* or both loci haplotypes further demonstrating that powdery mildew resistance is wide spread (Fig. 1). It would be interesting to collect more accessions of *V. piasezkii* from its native habitat in Northeast and Western China to carry out comparative disease evaluations aimed at identifying other accessions with similar or different loci. Such efforts would help to capture the maximum genetic diversity of powdery mildew resistance and potentially help to understand the mode of evolution of the resistance. It is also possible that both loci evolved independently of each other, and later combined into a single line with natural hybridization. In future studies, comparative genome sequence analysis of both the *Ren6* and *Ren7* loci could shed more light on the homology of the resistance genes and potentially explain the evolution of this powdery mildew resistance.

### Implication for breeding durable field resistance

Grape powdery mildew is a rapidly evolving pathogen as a result of its mixed reproductive strategies and strong selection pressure due to the extensive use of synthetic fungicides in all grape growing regions of the world [27]. Major *R* loci against powdery mildew have been identified in many North American, Central Asian and Chinese species [9, 14, 19, 21, 26]. In general, major genes confer a strong resistance against specific races of a pathogen and are stable across diverse environmental conditions. However, this monogenic resistance can create high selection pressure on the pathogen that could lead to the emergence of new virulent isolates [25, 74].

Durable disease resistance against pathogens such as powdery mildew is a primary objective of many grape breeding programs worldwide. A common theme among researchers is to adopt strategies to moderate selection pressure by combining or stacking *R* genes from different genetic sources and hence slow the evolution of virulent isolates and achieve durable resistance in the field. The identification of two powdery mildew *R* loci that segregate independently of each other is very important for grape breeders. To date, powdery mildew resistance loci have been identified and mapped on chromosomes 12, 13, 14, 15 and 18 from different native grape species and hybrids from North America, Central Asia and China [9–12, 14, 17, 19, 20, 34, 35]. The presence of *R* gene(s) on different chromosomes makes it easier to stack resistance via marker-assisted selection more effectively [24]. Current breeding strategies are also focused on combining *R* genes from different *Vitis* species with the assumption that they will have different recognition specificities [14]. This strategy is important to ensure that any mutation in a core effector will not lead to a loss of recognition by both R proteins simultaneously. At present, only limited information is available regarding the race-specificity of the different grapevine powdery mildew *R* loci. A recent study by Feechan et al. [14] demonstrated that the *Run1* and *Run2.1* loci, which originated from different breeding lines of *M. rotundifolia*, show clear differences in recognition of the *Run1*-breaking Musc4 isolate making them good candidates for stacking. Similarly, preliminary studies with *Ren6* and *Ren7* also suggest that the resistance conferred by these two loci is not compromised by the Musc4 isolate (Lance Cadle-Davidson, personal communication). In this regard, the addition of two new *R* loci from *V. piasezkii,* that we showed confer resistance to powdery mildew isolates from North America and Australia, probably evolved to resist isolates in China [15, 75], making these *R* loci a valuable addition to the repertoire of resistance loci for powdery mildew resistance breeding. *Vitis piasezkii*’s neutral fruit flavor and breeding compatibility with *V. vinifera* cultivars makes it ideal candidate to develop high quality resistant lines in a short interval of time. With the help of tightly linked markers, it will be possible to incorporate these *R* loci into advanced breeding lines that already have powdery mildew *R* loci incorporated from different sources to produce grapevines with durable resistance to this important pathogen.

## Conclusions

The Chinese grape species *V. piasezkii* is an excellent source of powdery mildew resistance. We developed a framework genetic map using an F1 breeding population with resistance from *V. piasezkii* DVIT2027. Data from multiple screens was used to identify and map two powdery mildew *R* loci designated *Ren6* and *Ren7* that reside on chromosome 9 and 19, respectively. Examination of the mapping population in multiple environments found that these loci conferred resistance to powdery mildew in different ways – *Ren6* provides a very rapid programmed cell death response, while *Ren7* is responsible for greatly restricting hyphal growth. These loci are very useful sources of resistance because they are located on different chromosomes than previously reported grape powdery mildew *R* loci. Stacking different *R* loci will help to breed new grape varieties with durable powdery mildew resistance and help prevent the adaptation of more virulent powdery mildew strains capable of overcoming resistant cultivars.

## Additional files

Additional file 1: Table S1. New SSR makers designed from the 12X genome sequence of PN40024 for chromosome 9 and 19. (DOCX 15 kb) Additional file 2: Table S2. Descriptive statistics of the phenotypic scores within the base mapping population 11-373. Powdery mildew symptoms in the field were evaluated in two subsequent years. Greenhouse, in vitro experiments and the qPCR-based molecular assay were carried out with three to four biological replicates of each seedling plant in 2014. (DOCX 14 kb) Additional file 3: Table S5. Two-way factorial Analysis of Variance with four powdery mildew isolates that were used to evaluate four genotypic classes of progeny plants with susceptible controls and parental genotypes. (DOCX 14 kb) Additional file 4: Table S3. A list of significantly distorted markers with *p* values. (DOCX 20 kb) Additional file 5: Figure S1. A framework genetic map of *Vitis piasezkii* DVIT2027. Markers that have significant segregation deviation from Mendelian ratios are marked with asterisks indicating the significance levels at alpha 0.01 = *, 0.05 = **, 0.001 = ***, 0.005 = ****, 0.0001 = *****, 0.0005 = ******, and 0.00001 = *******. (PNG 186 kb) Additional file 6: Table S4. QTLs detected in interval mapping (IM), multiple QTL mapping (MQM) in the base mapping population using different disease evaluation assays. The IM analysis was also carried out with genotypes possessing either *Ren6* (IM without *Ren7*) or *Ren7* (IM without Ren6) haplotypes. (DOCX 24 kb) Additional file 7: Figure S2. Gene and transposon annotation of the corresponding genomic region of *Ren6* (A) and *Ren7* (B) loci in the PN40024 sequence. The green color indicates the annotated genes labeled with gene ID and the maroon color is used for the mRNA of the corresponding gene. The blue color was used to mark the location of flanking markers on the sequence. All other colors indicate different types of transposable elements identified with the Repbase database. Transposable elements smaller than 100 bp were not included in the figure. (PNG 3624 kb)
