# Supplementary material for: Identification of two novel powdery mildew resistance loci, Ren6 and Ren7, from the wild Chinese grape species Vitis piasezkii
Source: BMC Plant Biol. 2016 Jul 29;16:170. doi: 10.1186/s12870-016-0855-8 (PMC4966781; doi:10.1186/s12870-016-0855-8)
Supplement: Additional file 1: Table S1. — New SSR makers designed from the 12X genome sequence of PN40024 for chromosome 9 and 19. (DOCX 15 kb) [file 12870_2016_855_MOESM1_ESM.docx]

**Supplemental Table 1 New SSR makers designed from the 12X genome sequence of PN40024 for chromosome 9 and 19**

| Marker Name | Forward Primer Sequence | Reverse Primer Sequence | Amplicon size |
| --- | --- | --- | --- |
| PN9-005 | TCAATTCACCTCCTCAAGGTTT | CTAGCAATCTTTTGGGCAGTTT | 238 |
| PN9-016 | CAACGATCACAGAAGGCAATAA | TGTGTATGGAAGACCAACTATGGA | 251 |
| PN9-042 | CTCCACCTTGGTTTGCTTATTC | ACAACAGCCTATGTTCCAGAGC | 366 |
| PN9-057 | GAGATGTTGTAGTGAAGATCAAGC | AGTAGGAAGAGATGTGGAAAAGAG | 190 |
| PN9-063 | TCCTAAGACAAAGTTCCCTTCA | GTTACAGTTGAATCCTTGCACA | 194 |
| PN9-066.1 | GCATCCTCTATGTCTTTATTTTGAGA | GATGGTTTGCACAAGCTAGAAA | 306 |
| PN9-067 | GGGTAGCAACATTGAAAAGGAC | AGAATTATCTTCGGCCCTCATT | 303 |
| PN9-067.2 | GTCAAGATAAAGCCTAAGTGGC | TGAGTAGATGTGACTACAATCCTTTG | 350 |
| PN9-068 | CCCCAATCTTAAATGGAACA | TAGTGGGACGTTGGACACTT | 182 |
| PN9-099 | TGCAACCATAATCAACGTGC | GCATGTGTAAACTAAGCCCTAACA | 272 |
| PN9-112 | TGTTAGCCCCATTTGGATT | CCATTTCAACATCTCTATTCCC | 242 |
| PN9-141 | GTTGGGGTCATGCAACTATCTT | ACAATGGGCTAGATTAAGCGAC | 383 |
| PN19-018 | CAAAACAAGTCCATTGCGTTTA | TGTCCCATGACAGTTTATCCAG | 197 |
| PN19-022 | TTTTATTTCCATTTTCTTTCTTTC | AGGAGAGATATTTTGCATTGAAG | 286 |
