# Supplementary material for: Identification of two novel powdery mildew resistance loci, Ren6 and Ren7, from the wild Chinese grape species Vitis piasezkii
Source: BMC Plant Biol. 2016 Jul 29;16:170. doi: 10.1186/s12870-016-0855-8 (PMC4966781; doi:10.1186/s12870-016-0855-8)
Supplement: Additional file 2: Table S2. — Descriptive statistics of the phenotypic scores within the base mapping population 11-373. Powdery mildew symptoms in the field were evaluated in two subsequent years. Greenhouse, in vitro experiments and the qPCR-based molecular assay were carried out with three to four biological replicates of each seedling plant in 2014. (DOCX 14 kb) [file 12870_2016_855_MOESM2_ESM.docx]

**Supplemental Table 2 Descriptive statistics for phenotypic scores of powdery mildew symptoms within the 11-373 grapevine mapping population**

| Phenotype Evaluation^a^ | Number | Mean | Variance | Min. | Median | Max. |
| --- | --- | --- | --- | --- | --- | --- |
| Leaf 2013 | 253 | 0.51 | 0.55 | 0 | 0.33 | 3.83 |
| Cane 2013 | 253 | 0.23 | 0.29 | 0 | 0 | 3.5 |
| Leaf 2014 | 261 | 1.29 | 3.09 | 0 | 0.5 | 5 |
| Cane 2014 | 261 | 0.88 | 2.41 | 0 | 0 | 5 |
| Greenhouse | 258 | 1.36 | 1.36 | 0 | 0.43 | 4 |
| *in vitro* | 258 | 1.55 | 2.57 | 0 | 0.97 | 4 |
| qPCR | 247 | 6.62 | 9.63 | 0.25 | 6.87 | 12.67 |

^a^ Powdery mildew symptoms in the field were evaluated in two subsequent years. Greenhouse, *in vitro* experiments and the qPCR-based molecular assay were carried out with three to four biological replicates of each seedling plant in 2014.
