# Supplementary material for: Identification of two novel powdery mildew resistance loci, Ren6 and Ren7, from the wild Chinese grape species Vitis piasezkii
Source: BMC Plant Biol. 2016 Jul 29;16:170. doi: 10.1186/s12870-016-0855-8 (PMC4966781; doi:10.1186/s12870-016-0855-8)
Supplement: Additional file 3: Table S5. — Two-way factorial Analysis of Variance with four powdery mildew isolates that were used to evaluate four genotypic classes of progeny plants with susceptible controls and parental genotypes. (DOCX 14 kb) [file 12870_2016_855_MOESM3_ESM.docx]

**Supplemental Table 5  Two-way factorial Analysis of Variance with four powdery mildew isolates that were used to evaluate four genotypic classes of progeny plants with susceptible controls and parental genotypes**

|  | Df | SS | MS | F | *p* Value |
| --- | --- | --- | --- | --- | --- |
| Genotypic Class | 3 | 111.58 | 37.19 | 40.835 | <2e-16 |
| Strain | 3 | 4.76 | 1.59 | 1.743 | 0.162 |
| Genotypic Class X Strain | 9 | 2.01 | 0.22 | 0.246 | 0.987 |
| Residuals | 108 | 98.37 | 0.91 |  |  |
