# Supplementary material for: Identification of two novel powdery mildew resistance loci, Ren6 and Ren7, from the wild Chinese grape species Vitis piasezkii
Source: BMC Plant Biol. 2016 Jul 29;16:170. doi: 10.1186/s12870-016-0855-8 (PMC4966781; doi:10.1186/s12870-016-0855-8)
Supplement: Additional file 4: Table S3. — A list of significantly distorted markers with p values. (DOCX 20 kb) [file 12870_2016_855_MOESM4_ESM.docx]

**Supplemental Table 3. A list of significantly distorted markers with *p* values**

| Chromosome | Locus | Segregation | χ2 | Df | *p* Value |
| --- | --- | --- | --- | --- | --- |
| 1 | VVS29 | <abxcd> | 8.09 | 3 | ** |
| 1 | VMC8a7 | <abxcd> | 7.89 | 3 | ** |
| 1 | AF378125 | <nnxnp> | 10.37 | 1 | *** |
| 1 | VVIq57 | <abxcd> | 13.54 | 3 | *** |
| 1 | ctg1010271 | <efxeg> | 11.97 | 3 | *** |
| 1 | VMCNg2g7 | <nnxnp> | 15.36 | 1 | ****** |
| 1 | ctg1008034 | <nnxnp> | 15.48 | 1 | ****** |
| 1 | VMCNg1h7 | <abxcd> | 17.53 | 3 | **** |
| 1 | ctg1025664 | <abxcd> | 19.91 | 3 | ***** |
| 1 | ctg1011774 | <abxcd> | 14.16 | 3 | *** |
| 1 | VMC7g5 | <efxeg> | 15.56 | 3 | *** |
| 1 | VVIs21 | <abxcd> | 16.36 | 3 | **** |
| 1 | VMC2b3 | <abxcd> | 16.66 | 3 | **** |
| 1 | ctg1026392 | <nnxnp> | 10.52 | 1 | *** |
| 1 | VVIf52 | <nnxnp> | 6.07 | 1 | ** |
| 1 | VMC9d3 | <efxeg> | 10.9 | 3 | ** |
| 4 | VMC2b5 | <abxcd> | 7.88 | 3 | ** |
| 5 | SCA20-5 | <efxeg> | 8.11 | 3 | ** |
| 5 | VVIv21 | <abxcd> | 11.24 | 3 | ** |
| 5 | VMC16d4 | <efxeg> | 8.04 | 3 | ** |
| 6 | VMC5c5 | <nnxnp> | 7.33 | 1 | *** |
| 6 | VMC2h9 | <nnxnp> | 7.45 | 1 | *** |
| 7 | VMC7a4 | <efxeg> | 8.85 | 3 | ** |
| 8 | VVIv15.2 | <abxcd> | 10.91 | 3 | ** |
| 8 | VMC7h2 | <nnxnp> | 11.97 | 1 | **** |
| 8 | UDV126 | <nnxnp> | 14.65 | 1 | ***** |
| 9 | LG9-sc2014 | <abxcd> | 8.95 | 3 | ** |
| 9 | CD009354 | <abxcd> | 9.12 | 3 | ** |
| 11 | VVIm04 | <nnxnp> | 8.41 | 1 | *** |
| 11 | VMC3e12 | <abxcd> | 8.44 | 3 | ** |
| 14 | VVC62 | <abxcd> | 8.99 | 3 | ** |
| 14 | VMCNg1e1 | <abxcd> | 9.37 | 3 | ** |
| 16 | UDV086 | <abxcd> | 49.34 | 3 | ****** |
| 19 | UDV114 | <efxeg> | 12.13 | 3 | *** |

^a^ Markers that have significant segregation deviation from the Mendelian ratios are marked with asterisks indicating the significance levels at alpha 0.01=*, 0.05=**, 0.001=***, 0.005=****, 0.0001=*****, 0.0005=******, 0.00001=*******.
