# Supplementary figures and images for: Identification of two novel powdery mildew resistance loci, Ren6 and Ren7, from the wild Chinese grape species Vitis piasezkii
Source: BMC Plant Biol. 2016 Jul 29;16:170. doi: 10.1186/s12870-016-0855-8 (PMC4966781; doi:10.1186/s12870-016-0855-8)

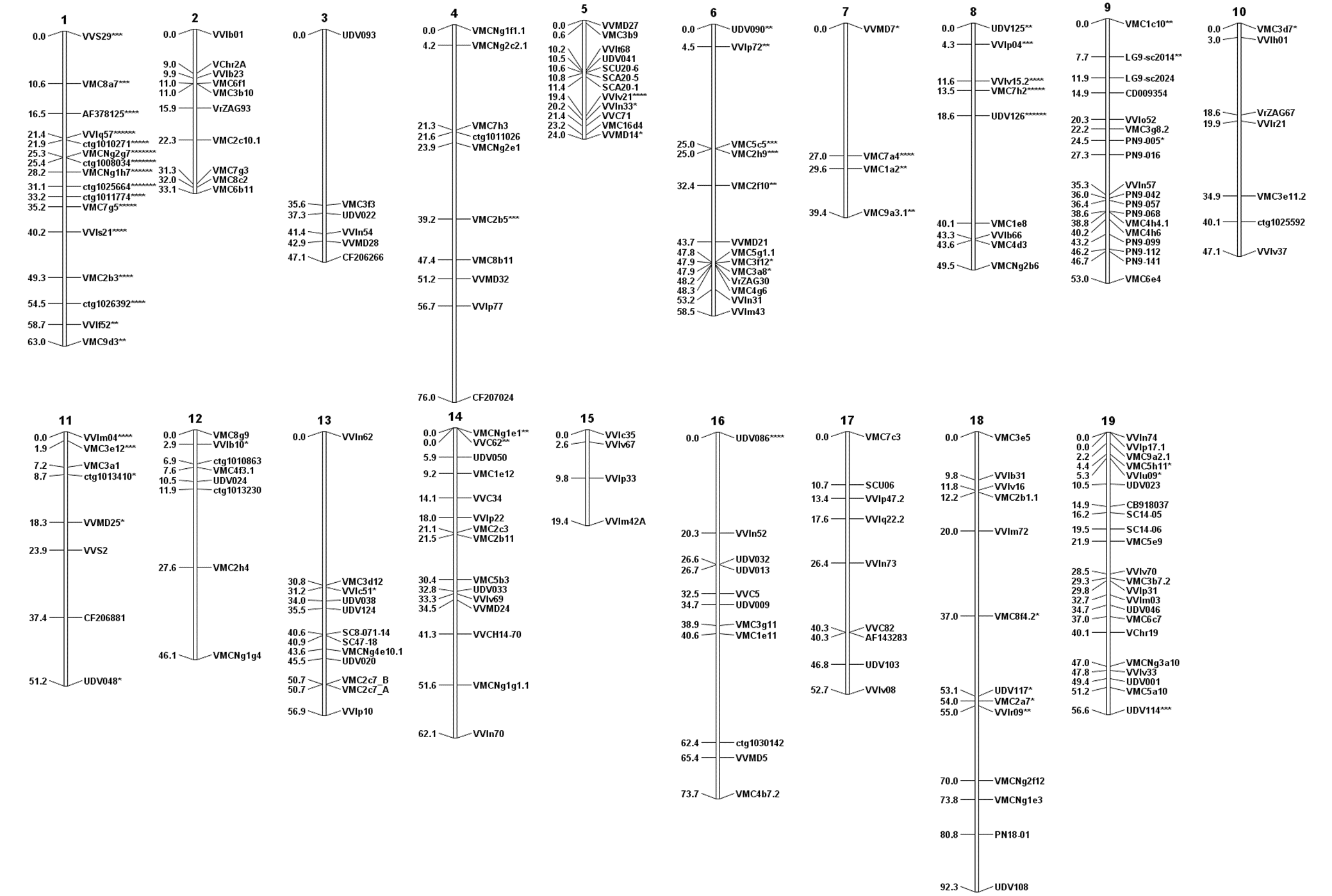

Supplement: Additional file 5: Figure S1. — A framework genetic map of Vitis piasezkii DVIT2027. Markers that have significant segregation deviation from Mendelian ratios are marked with asterisks indicating the significance levels at alpha 0.01 = *, 0.05 = **, 0.001 = ***, 0.005 = ****, 0.0001 = *****, 0.0005 = ******, and 0.00001 = *******. (PNG 186 kb) [file 12870_2016_855_MOESM5_ESM.png]

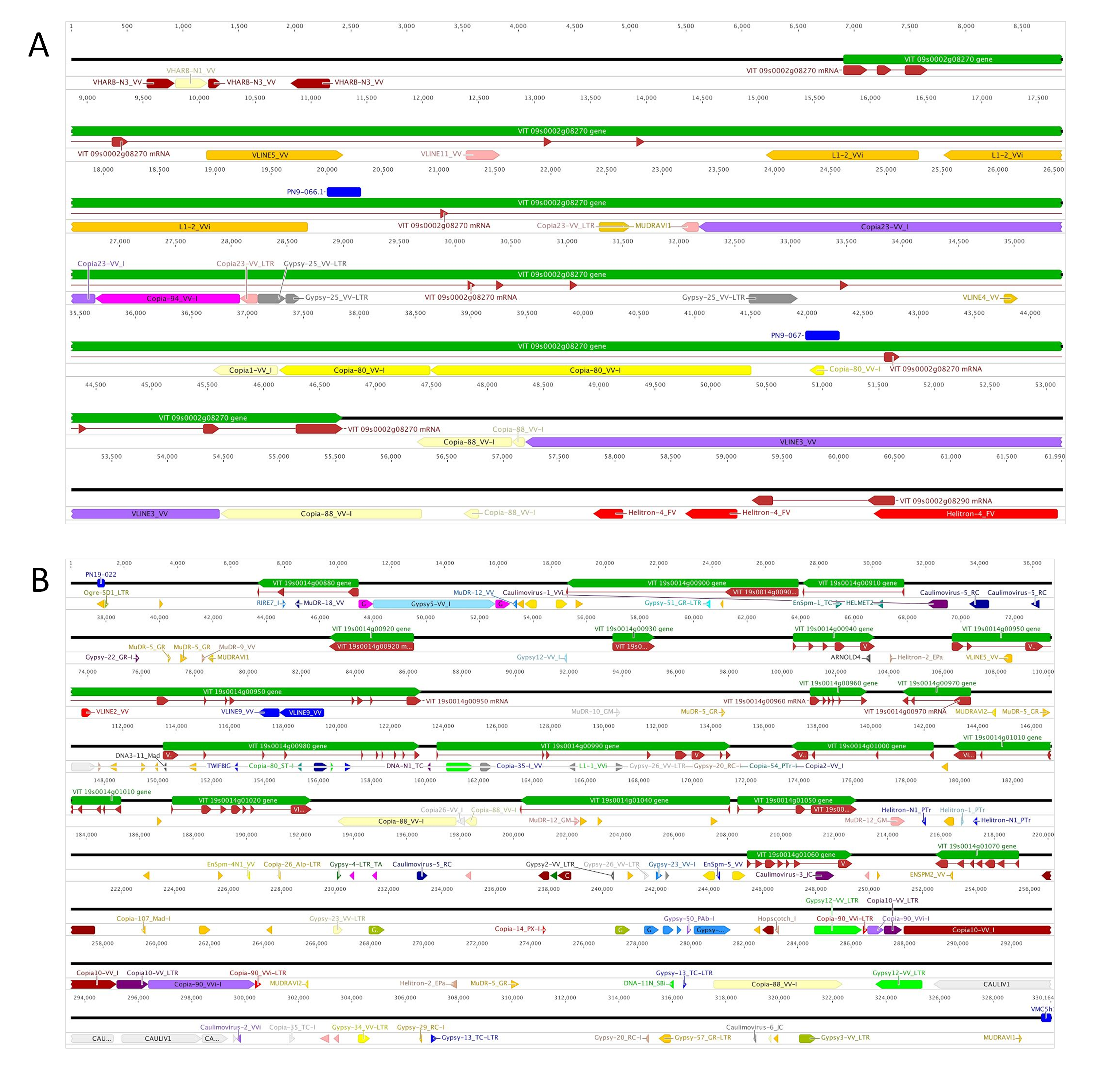

Supplement: Additional file 7: Figure S2. — Gene and transposon annotation of the corresponding genomic region of Ren6 (A) and Ren7 (B) loci in the PN40024 sequence. The green color indicates the annotated genes labeled with gene ID and the maroon color is used for the mRNA of the corresponding gene. The blue color was used to mark the location of flanking markers on the sequence. All other colors indicate different types of transposable elements identified with the Repbase database. Transposable elements smaller than 100 bp were not included in the figure. (PNG 3624 kb) [file 12870_2016_855_MOESM7_ESM.png]
