# Supplementary material for: Identification of two novel powdery mildew resistance loci, Ren6 and Ren7, from the wild Chinese grape species Vitis piasezkii
Source: BMC Plant Biol. 2016 Jul 29;16:170. doi: 10.1186/s12870-016-0855-8 (PMC4966781; doi:10.1186/s12870-016-0855-8)
Supplement: Additional file 6: Table S4. — QTLs detected in interval mapping (IM), multiple QTL mapping (MQM) in the base mapping population using different disease evaluation assays. The IM analysis was also carried out with genotypes possessing either Ren6 (IM without Ren7) or Ren7 (IM without Ren6) haplotypes. (DOCX 24 kb) [file 12870_2016_855_MOESM6_ESM.docx]

**Supplemental Table 4 QTLs detected with interval mapping (IM), multiple QTL mapping (MQM) in the base mapping population using different disease evaluation assays**

| Phenotype | Mapping Method | Chromosome | LOD Max | Genome Wide Threshold, 95% | Variance Explained (%) | Phenotypic Variance | Location on the Map (cM) | Flanking Markers | | Cofactor(s) |
| --- | --- | --- | --- | --- | --- | --- | --- | --- | --- | --- |
| Leaf 2013 | IM | 9 | 9.95 | 2.70 | 16.6 | 0.56 | 38.613 | PN9-057 | PN9-068 | - |
| Cane 2013 | IM | 9 | 3.17 | 2.60 | 5.6 | 0.29 | 38.613 | PN9-057 | PN9-068 | - |
| Leaf 2014 | IM | 9 | 31.78 | 2.80 | 42.9 | 3.09 | 38.423 | PN9-057 | PN9-068 | - |
| Cane 2014 | IM | 9 | 14.05 | 2.80 | 22.0 | 2.41 | 38.613 | PN9-057 | PN9-068 | - |
| Greenhouse | IM | 9 | 50.60 | 2.80 | 59.5 | 2.62 | 38.423 | PN9-057 | PN9-068 | - |
| *in vitro* | IM | 9 | 54.30 | 2.80 | 61.9 | 2.57 | 38.423 | PN9-057 | PN9-068 | - |
| qPCR | IM | 9 | 37.60 | 2.80 | 50.5 | 9.63 | 38.423 | PN9-057 | PN9-068 | - |
| Leaf 2013 | IM | 19 | 2.79 | 2.70 | 4.9 | 0.56 | 2.006 | VVIp17.1 | VMC9a2.1 | - |
| Cane 2013 | IM | 19 | 3.05 | 2.60 | 5.4 | 0.29 | 1.006 | VVIp17.1 | VMC9a2.1 | - |
| Leaf 2014 | IM | 19 | 11.38 | 2.80 | 18.2 | 3.09 | 1.006 | VVIp17.1 | VMC9a2.1 | - |
| Cane 2014 | IM | 19 | 11.92 | 2.80 | 19.0 | 2.41 | 1.006 | VVIp17.1 | VMC9a2.1 | - |
| Greenhouse | IM | 19 | 9.03 | 2.80 | 14.9 | 2.62 | 3.24 | VMC9a2.1 | VMC5h11 | - |
| *in vitro* | IM | 19 | 6.58 | 2.80 | 11.1 | 2.57 | 3.24 | VMC9a2.1 | VMC5h11 | - |
| qPCR | IM | 19 | 5.44 | 2.80 | 9.7 | 9.63 | 2.006 | VMC9a2.1 | VMC5h11 | - |
| Leaf 2013 | MQM | 9 | 8.01 | 2.70 | 13.0 | 0.56 | 36.055 | PN9-042 | PN9-057 | VVIn57, PN9-042 |
| Cane 2013 | MQM | 9 | 9.55 | 2.60 | 15.4 | 0.29 | 36.423 | PN9-057 | PN9-068 | VVIn57, PN9-042 |
| Leaf 2014 | MQM | 9 | 40.43 | 2.80 | 42.8 | 3.09 | 38.423 | PN9-057 | PN9-068 | PN9-068 |
| Cane 2014 | MQM | 9 | 17.09 | 2.80 | 21.2 | 2.41 | 38.423 | PN9-057 | PN9-068 | PN9-068 |
| Greenhouse | MQM | 9 | 67.40 | 2.80 | 60.1 | 2.62 | 38.423 | PN9-057 | PN9-068 | PN9-068 |
| *in vitro* | MQM | 9 | 66.28 | 2.80 | 62.0 | 2.57 | 38.423 | PN9-057 | PN9-068 | PN9-068 |
| qPCR | MQM | 9 | 44.95 | 2.80 | 52.0 | 9.63 | 38.423 | PN9-057 | PN9-068 | PN9-068 |
| Leaf 2013 | MQM | 19 | 3.55 | 2.70 | 5.0 | 0.56 | 5.357 | VVIu09 | VMC5h11 | VVIu09 |
| Cane 2013 | MQM | 19 | 3.18 | 2.60 | 4.6 | 0.29 | 0.006 | VVIp17.1 | VMC9a2.1 | - |
| Leaf 2014 | MQM | 19 | 18.56 | 2.80 | 16.0 | 3.09 | 5.357 | VVIu09 | VMC5h11 | VVIu09 |
| Cane 2014 | MQM | 19 | 14.99 | 2.80 | 18.1 | 2.41 | 1.006 | VVIp17.1 | VMC9a2.1 | VVIn74 |
| Greenhouse | MQM | 19 | 25.18 | 2.80 | 14.7 | 2.62 | 5.357 | VVIu09 | VMC5h11 | VVIu09 |
| *in vitro* | MQM | 19 | 18.38 | 2.80 | 10.7 | 2.57 | 5.357 | VVIu09 | VMC5h11 | VVIu09 |
| qPCR | MQM | 19 | 12.71 | 2.80 | 10.5 | 9.63 | 3.24 | VMC9a2.1 | VMC5h11 | VMC5h11 |
| Leaf 2013 | IM wo*Ren7* | 9 | 10.09 | 2.90 | 28.6 | 0.79 | 38.613 | PN9-057 | PN9-068 | - |
| Cane 2013 | IM wo*Ren7* | 9 | 2.97 | 2.90 | 9.4 | 0.46 | 38.613 | PN9-057 | PN9-068 | - |
| Leaf 2014 | IM wo*Ren7* | 9 | 48.76 | 2.80 | 79.2 | 3.98 | 38.613 | PN9-057 | PN9-068 | - |
| Cane 2014 | IM wo*Ren7* | 9 | 16.03 | 2.90 | 40.3 | 3.39 | 38.613 | PN9-057 | PN9-068 | - |
| Greenhouse | IM wo*Ren7* | 9 | 95.76 | 2.80 | 95.4 | 3.31 | 38.423 | PN9-057 | PN9-068 | - |
| *in vitro* | IM wo*Ren7* | 9 | 69.91 | 2.90 | 89.6 | 3.36 | 38.613 | PN9-057 | PN9-068 | - |
| qPCR | IM wo*Ren7* | 9 | 36.51 | 2.80 | 71.2 | 11.10 | 38.423 | PN9-057 | PN9-068 | - |
| Leaf 2013 | IM wo*Ren6* | 19 | 4.66 | 2.90 | 15.9 | 0.85 | 2.006 | VVIp17.1 | VMC9a2.1 | - |
| Cane 2013 | IM wo*Ren6* | 19 | 2.76 | 3.10 | 9.7 | 0.53 | 1.006 | VVIp17.1 | VMC9a2.1 | - |
| Leaf 2014 | IM wo*Ren6* | 19 | 26.26 | 2.80 | 60.8 | 3.45 | 2.006 | VVIp17.1 | VMC9a2.1 | - |
| Cane 2014 | IM wo*Ren6* | 19 | 14.44 | 2.80 | 40.3 | 3.43 | 1.006 | VVIp17.1 | VMC9a2.1 | - |
| Greenhouse | IM wo*Ren6* | 19 | 35.58 | 2.80 | 71.9 | 2.08 | 3.24 | VMC9a2.1 | VMC5h11 | - |
| *in vitro* | IM wo*Ren6* | 19 | 28.09 | 2.80 | 63.3 | 1.80 | 3.24 | VMC9a2.1 | VMC5h11 | - |
| qPCR | IM wo*Ren6* | 19 | 15.27 | 2.80 | 43.3 | 6.63 | 2.24 | VMC9a2.1 | VMC5h11 | - |

^a^ The IM analysis was also carried out with genotypes possessing either *Ren6* (IM without *Ren7*) or *Ren7* (IM without *Ren6*) haplotypes.
